# Hydrophobic Interaction: A Promising Driving Force for the Biomedical Applications of Nucleic Acids

**DOI:** 10.1002/advs.202001048

**Published:** 2020-07-01

**Authors:** Fan Xiao, Zhe Chen, Zixiang Wei, Leilei Tian

**Affiliations:** ^1^ Department of Materials Science and Engineering Southern University of Science and Technology 1088 Xueyuan Blvd. Nanshan District Shenzhen Guangdong 518055 P. R. China; ^2^ School of Materials Science and Engineering Harbin Institute of Technology Nangang District Harbin 150001 P. R. China; ^3^ Cancer Centre and Centre of Reproduction Development and Aging Faculty of Health Sciences University of Macau Taipa Macau 999078 P. R. China

**Keywords:** biomedicine, biosensors, functional nucleic acids, hydrophobic interactions

## Abstract

The comprehensive understanding and proper use of supramolecular interactions have become critical for the development of functional materials, and so is the biomedical application of nucleic acids (NAs). Relatively rare attention has been paid to hydrophobic interaction compared with hydrogen bonding and electrostatic interaction of NAs. However, hydrophobic interaction shows some unique properties, such as high tunability for application interest, minimal effect on NA functionality, and sensitivity to external stimuli. Therefore, the widespread use of hydrophobic interaction has promoted the evolution of NA‐based biomaterials in higher‐order self‐assembly, drug/gene‐delivery systems, and stimuli‐responsive systems. Herein, the recent progress of NA‐based biomaterials whose fabrications or properties are highly determined by hydrophobic interactions is summarized. 1) The hydrophobic interaction of NA itself comes from the accumulation of base‐stacking forces, by which the NAs with certain base compositions and chain lengths show properties similar to thermal‐responsive polymers. 2) In conjugation with hydrophobic molecules, NA amphiphiles show interesting self‐assembly structures with unique properties in many new biosensing and therapeutic strategies. 3) The working‐mechanisms of some NA‐based complex materials are also dependent on hydrophobic interactions. Moreover, in recent attempts, NA amphiphiles have been applied in organizing macroscopic self‐assembly of DNA origami and controlling the cell–cell interactions.

## Graphical abstract

Herein, recent advances in nucleic acid‐based biomaterials whose fabrications or properties are highly dependent on hydrophobic interactions are summarized. Such biomaterials exhibit emerging properties, such as diversified functionalities, more sensitive stimuli‐responsiveness, and hierarchical self‐assembly capability, which determines that hydrophobic interaction will become a potent tool in the design of nucleic acid‐based biomaterials in the future.

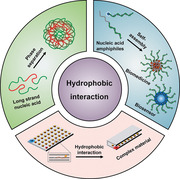

## 1 Introduction

Nucleic acids (NAs) are macromolecules with well‐defined molecular structures; the combination of the four nucleotides (A, T, G, C) can precisely encode unlimited information, which is the most attractive feature of NAs. Unlike synthesized polymer, which is comprised of structure‐determined repeat units, the functional unit of NAs is a strand of sequence. Instead of structural design, the functionality of NAs is screened and optimized from different orders and combinations of nucleotides. Therefore, a reliable database of NA sequence‐functionality has been quickly established.^[^ ^1^, ^2^ ^]^ As NA solid‐phase synthesis technology has become mature, any functional sequence (<100 nucleotides) will be easy to get, which makes NAs a highly accessible material.^[^ ^3^, ^4^ ^]^ In addition to the easy manipulation, NA also shows numerous biomedical functionalities, good biocompatibility, and enriched chemistry, and all of these features make NA an indispensable material for nanomedicine design.^[^ ^5^, ^6^ ^]^ 1) First, due to the recognition and responsiveness of NA, it has been broadly applied in biosensing and ‐imaging.^[^ ^7^ ^]^ NA can recognize its complementary sequence and induce conformational changes or strand displacements to generate signal output. Not limited to base‐pairing interactions, the cytosine‐rich DNA sequence can recognize pH variation and form quadruplex structure with intercalated C—C^+^ interactions, which has become an important building block for the design of pH‐biosensors.^[^ ^8^ ^]^ In addition to H^+^, many metal ions, such as K^+^ (K^+^‐centered G‐quadruplet), Ag^+^ (C—Ag^+^—C), Hg^2+^ (T—Hg^2+^—T), and Cu^+^ (C—Cu^+^—C), can form special intercalation structures with certain bases. Therefore, the conformational change of NA will drive signal transduction for the detection of these metal ions. Much more than all of these, the recognition capability of NA can be extended to no limitations on targets upon the development of aptamers by systematic evolution of ligands by exponential enrichment (SELEX) technique.^[^ ^9^ ^]^ Aptamers are single‐stranded DNA or RNA strands that can bind to targets with high affinity and specificity by folding into certain conformations. To date, DNA and RNA aptamers have been widely used to construct biosensors and diagnostics through identifying various targets, up to bacteria and cell, protein, peptide, amino acid, and down to small molecule and metal ion. 2) Many NAs also show therapeutic functions, which can specifically inhibit the function of a particular gene involved in disease,^[^ ^6^ ^]^ mainly including antisense oligonucleotides (ASOs) and small interfering RNAs (siRNAs). ASOs are single‐strand DNAs (ssDNAs) in 8–50 nt lengths, which bind with mRNA to block function or initiate degradation by endogenous Ribonuclease H (RNase H). On the other side, siRNA is a 20–28 nt long double‐strand DNA (dsDNA), which suppress gene expression through activating the RNA‐induced silencing complex (RISC) and result in target mRNA degradation. In addition to gene regulation and therapy, unmethylated cytosine‐phosphate‐guanine (CpG) motif‐containing DNA (CpG DNA) is also used as immunostimulants. As a toll‐like receptor 9 (TLR9) agonist, CpG DNA can generate immune responses in cancer therapy.^[^ ^10^, ^11^ ^]^

Although NA shows many interesting biomedical functions, its effective pharmacological use is still facing challenges. NA is unstable in the bloodstream and rapidly cleared by the body, can induce immunogenicity, and lack cell membrane permeability.^[^ ^6^, ^12^ ^]^ Therefore, different strategies have been developed to make NAs an applicable material. All the strategies are based on the idea of densely packing NAs to increase biostability and cell uptake capability, which can be realized by covalent and noncovalent approaches. An ssDNA comprises hydrophobic bases and negatively charged phosphate‐sugar backbone. Thus the available interactions of DNA include the hydrogen bonding between nucleotides that can drive complementary strands together, the electrostatic attraction between the negatively charged DNA and cations or positively charged molecules, and the coordination interactions between DNA bases and metal ions.^[^ ^13^ ^]^ Moreover, by the matured synthesis method, DNA can be easily modified with a lot of functional groups for further conjugation reactions.

1) Electrostatic interactions are the electric force between any two charged molecules. As DNA is a negatively charged polyelectrolyte, multivalent cations become the most‐used nonviral transfection vectors for gene therapy,^[^ ^14^, ^15^ ^]^ including cationic lipids, short‐chain polyamides (e.g., spermine and spermidine), natural and synthetic poly(amino acids), cationic water‐soluble polymers (e.g., linear and branched polyethyleneimine), and cationic amphiphilic polymers. This method is efficient and straightforward, but it remains some limitations. For instance, generally, cationic polymers show low condensation efficiencies to short NAs, such as siRNA and ASO. Also, to construct a stable polyplex, the charge ratio between the cationic polymer and DNA should be larger than 10, and the excessive amount of cationic polymer will result in high cytotoxicity.^[^ ^16^ ^]^ The strong interaction between the two charged polymers will ensure the biostability of DNA during the blood circulation. However, the resultant polyplex generally lacks the stimuli‐responsive capability, resulting in difficulties in the proper release of therapeutic NAs at the cytoplasm or nucleus.
2) The hydrogen‐bonding interactions following the elegant Watson–Crick base‐pairing rule have been widely applied in the fabrication of nanostructures with precise dimensions and smart properties, which makes DNA become the most useful building material in nanoscience and technology.^[^ ^17^, ^18^ ^]^ The most representative technique is DNA Origami, which fabricates nanostructures by utilizing hundreds of short single strands (staple strands) to fold an M13 phage DNA into the desired structures.^[^ ^19^ ^]^ Recently, Ding and co‐workers utilized origami technique to construct an autonomous DNA robot, which could deliver thrombin specifically to tumor‐associated blood vessels and induce intravascular thrombosis, resulting in tumor necrosis and inhibition of tumor growth.^[^ ^20^ ^]^ This work proved that it is potential to apply the DNA origami technique in precise and smart drug delivery for cancer therapy. However, the disadvantages, such as the complicated fabrication process and the high cost, will also limit its practical applications.
3) The coordinative or electrostatic interactions between the nucleobases/phosphate backbones of DNAs and metal ions provide another approach to fabricate functional DNA‐metal hybrid nanomaterials.^[^ ^21^ ^]^ On the one hand, the DNA sequences act as nucleation sites and the stabilizing ligands for metal nanoparticles. On the other hand, the metal components will endow more functionalities to the DNA‐metal hybrid nanomaterials, promoting their applications in the biological/chemical detecting, cellular and in vivo imaging, and therapeutics. Recently, Li and co‐workers utilized the coordination‐driven self‐assembly of Fe^2+^ ions and DNAs to produce DNA nanostructures with well‐controlled morphologies and functionalities.^[^ ^22^ ^]^ The DNA–Fe hybrid nanomaterials showed a high cellular permeability, which could efficiently deliver functional DNAs to cells and showed a high in vivo therapeutic efficacy.
4) DNA can be conjugated to the surface of other nanomaterials through the functional groups that are chemically modified at the ends of DNA strands, which can also efficiently protect DNA from enzymatic cleavage and enhance the cell permeability.^[^ ^23^, ^24^, ^25^, ^26^, ^27^ ^]^ The most famous example is the conjugation between the thiol‐modified DNAs and gold nanoparticles (AuNPs), which is a very reactive and efficient reaction. On the one hand, AuNP is a very efficient fluorescence quencher, which can combine with the molecular recognition properties of DNA to develop “nanoflares” for intramolecular imaging and sensing applications. Nowadays, AuNP–DNA conjugates have become a very ideal platform for the design of biosensors. On the other hand, DNA strands are densely conjugated on the surface of AuNPs to form a closely packed and orientated DNA shell. The “cluster effect” of DNA makes the AuNP–DNA structures (which are called spherical nucleic acids in some studies) show some distinctive properties, such as an enhanced nuclease resistance, a higher cellular uptake capability, and which even show brain–blood barrier crossing capability.^[^ ^25^, ^26^ ^]^ Therefore, these properties also make AuNP–DNA structures perfect systems for gene delivery.

**Figure 1:**
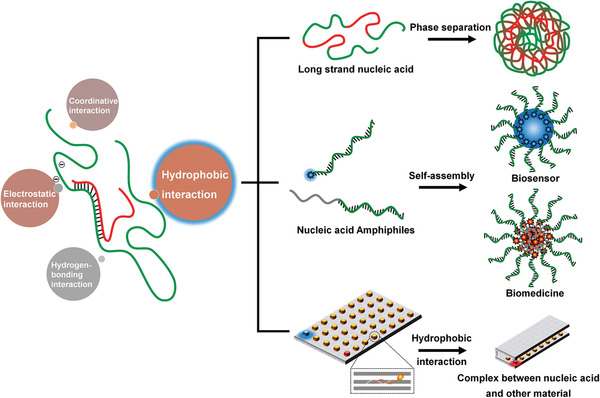
Schematic illustration of hydrophobic interaction as a promising driving force for the biomedical applications of nucleic acid‐based biomaterials.

Except for the above‐mentioned methods, hydrophobic interactions may provide another possible force to construct DNA‐based nanomaterials for biomedical applications. Hydrophobic interaction, also known as hydrophobic effect, is a kind of property of nonpolar molecules (or hydrophobic moieties of amphiphiles), which can drive these molecules to assemble to form anhydrous domains in aqueous solution. Essentially, the source of the hydrophobic effect is the entropy effect caused by nonpolar solutes destroying hydrogen bonds between water molecules.^[^ ^28^, ^29^ ^]^ In biophysics, hydrophobic interactions play an essential role in the 3D structure of proteins.^[^ ^30^ ^]^ For a globular protein, its surface is usually surrounded by a layer of hydrophilic residues in aqueous solution, and residues with hydrophobic side chains are usually inside the protein. As for the synthetic amphiphilic block‐copolymers, hydrophobic interaction is also very important in its assembly; as the controlled “living” polymerization technique has been well developed, the molecular weights of the hydrophobic and hydrophilic blocks can be precisely tuned to control the degree of hydrophobic interactions, by which the block copolymers can self‐assemble into nanoarchitectures with various sizes, morphologies, and smart stimuli‐responsive properties, making them distinctive in drug/gene delivery applications.^[^ ^31^, ^32^, ^33^ ^]^ Regarding constructing DNA‐based biomaterials through hydrophobic interactions shows several advantages. 1) Hydrophobic interaction can stabilize DNA to enhance its biostability. 2) Hydrophobic interactions can enhance the interaction between DNA and other nanomaterials to fabricate functional nanocomposites. 3) Hydrophobic interaction is dynamic and highly adjustable, which will allow the desired materials to respond more sensitively to environmental stimuli, enabling the design of smarter biomaterials. 4) Higher‐order self‐assembly structures will be constructed by introducing hydrophobic interaction into the structural DNA assembly. Intrinsically, DNA is a polymer whose molecular weight and conformation can be well‐defined by the sequence. Therefore, DNA shows overwhelming advantages in the accurate control of hydrophobic interactions for further self‐assembly. For one thing, DNA shows some amphiphilic nature as its base is hydrophobic, and its phosphate backbone is hydrophilic. Hence the length change, hybridization/dehybridization, and conformation change (e.g., i‐motif and G‐quadruplex) of DNA can influence the hydrophobicity of DNA and eventually affect the assembly behavior of DNA‐based materials. The hydrophobic interaction will not only affect the condensation state of DNA and also play an important role in determining the properties of its complex with other nanomaterials.^[^ ^34^, ^35^ ^]^ For another, DNA can be used as the hydrophilic block to synthesize DNA‐based amphiphilic block copolymer, and the resultant self‐assemblies can well reserve the functionality of DNA and generate many new properties owing to the hydrophobic interactions.^[^ ^36^, ^37^, ^38^, ^39^, ^40^, ^41^ ^]^ In this regard, the hydrophobicity of the hydrophobic block can also be tuned to control the properties of DNA‐based amphiphilic block copolymer. Due to the interesting and important role of hydrophobic interactions in DNA‐based materials, in this review, we will summary the recently developed strategies that mainly use hydrophobic interactions to construct DNA‐based biomedical materials (**Figure** **1**), including 1) hydrophobic–hydrophilic phase separation for intrinsic DNA condensation; 2) biomedical materials based on the self‐assembly of nucleic acid amphiphiles; and 3) biomedical materials based on the hydrophobic‐interaction‐stabilized complexes between DNA and other nanomaterials.

## 2 Hydrophobic Interaction from Base‐Stacking in Pure Nucleic Acid

Many investigations have demonstrated that the DNA double helix is mainly stabilized by coin‐pile stacking of base pairs and less by the hydrogen bonding between matched bases that most textbooks still refer to.^[^ ^34^, ^35^, ^42^ ^]^ Stacked bases attract to one another through Van der Waals forces; the energy associated with a single interaction has a little significance to the overall DNA structure, however, the net effect summed over the numerous ones, results in substantial stability. Therefore, the hydrophobic base stacking has been considered as the primary contributor to DNA double‐helix stability. A recent study has revealed that the hydrophobic interaction of DNA plays an important role in biological activity, which may have catalytic roles to activate DNA polymerase.^[^ ^35^ ^]^ As a result, instead of utilizing hydrogen‐bonding base‐pairing, the hydrophobic interaction is also necessary to construct DNA nanostructures for biomedical applications.

Recently, an interesting phenomenon has been observed from rolling circle amplification (RCA), in which a part of the RCA product is directly converted to flower‐like microsized particles after the reaction.^[^ ^43^, ^44^, ^45^ ^]^ Motivated by this observation, low‐cost functional DNA materials fabricated by RCA was put forward for its superior biostability endowed by its densely compacted flower‐like DNA nano/microstructure. RCA is a classic isothermal amplification technique, which can efficiently produce a high quantity of long ssDNA (lssDNA); the sequence of the lssDNA is well defined by and repeatedly copied from the circular template. As a result, multiple functional sites are polymerized together into an RCA product. It has been investigated that the flower‐like DNA structure was formed by the twine of long flexible RCA products, the process of which was induced by the high concentration of magnesium pyrophosphate (MgPPi), a byproduct of RCA reaction.^[^ ^44^, ^46^ ^]^

**Figure 2:**
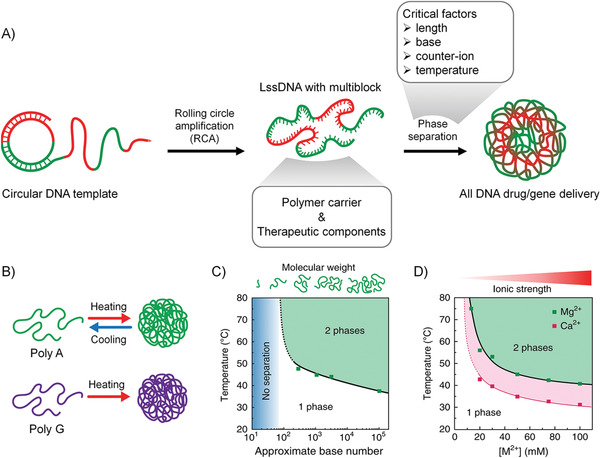
A) Illustration for hydrophobic–hydrophilic phase separation of lssDNA produced by rolling circle amplification. B) Illustration for heat‐induced phase separation of purine‐rich lssDNA. C) A phase diagram of the cloud point temperature corresponding to the length of DNA at [Mg^2+^] = 50 × 10^−3^ m. D) A phase diagram of the cloud point temperature of poly A (≈1000‐base long) corresponding to the concentration of the counter‐ions. The panels (B)–(D) are reproduced with permission.^[^ ^47^ ^]^ Copyright 2018, Nature Publishing Group.

According to our investigations, other than MgPPi, the addition of an appropriate concentration of Mg^2+^ could also condense the RCA product and induce the formation of a similar DNA structure with biostability against enzymatic degradation.^[^ ^21^ ^]^ The RCA nanoparticles condensed by excessive Mg^2+^ showed a smaller size of ≈100 nm, which can still keep intact and stable after going through the desalting column. There are three critical factors for the condensation of the RCA products and the subsequent biomedical applications. 1) The aromatic structure of the DNA base is hydrophobic, and the phosphate backbone is hydrophilic. A DNA strand can be well dissolved in aqueous solution without phase separation because the charge of its phosphate backbone allows the DNA strands to repel each other and achieves a good dispersion. Therefore, for an efficient condensation, a high concentration of counter‐ions is required to screen the surface charge of DNA. 2) Oligonucleotides with low molecular weights cannot be condensed by Mg^2+^, which suggested that the polymer nature of the enzymatically synthesized lssDNA is essential for the condensation process. Generally, compared with small molecules, polymers show stronger intramolecular interactions and more chain flexibility. Therefore, only a long DNA strand with significant hydrophobic base stacking can go through hydrophobic–hydrophilic phase separation. 3) Poor biostability is the biggest obstacle for the practical application of therapeutic NAs. For biomedical applications, the RCA technique can polymerize multiple strands of functional NAs to yield lssDNA with super‐high molecular weight. Also, the produced lssDNA shows a highly increased tendency to form condensed structures with high biostability. As a result, the lssDNAs produced by RCA can act as both the polymer carrier and therapeutic NAs to construct self‐delivered all‐DNA nanomedicines for chemo‐/gene therapy (**Figure** **2**A).

In another research, Walther et al. demonstrated that the RCA products show physical properties similar to thermoresponsive polymers, which would go through hydrophobic–hydrophilic phase separation under a certain temperature. Also, they studied the key factors that will affect the phase separation process.^[^ ^47^ ^]^ First, they found that only purine‐rich lssDNA (Poly A and Poly G) exhibited heat‐induced phase separation in the presence of 50 × 10^−3^ m Mg^2+^ (Figure 2B). In contrast, pyrimidine‐rich lssDNA did not show phase separation. Second, the phase separation due to hydrophobic interaction depends on the degree of polymerization of the DNA. The authors observed that as the length of the lssDNA increases, the cloud point temperature (i.e., the temperature at which phase separation occurs) decreases (Figure 2C), which may be due to the increased length of ssDNA, resulting in a stronger hydrophobic interaction that makes phase separation easier. It is worth noting that when the length of DNA is shorter than ≈100 nucleobases, the DNA remains in a dissolved state regardless of the temperature. Finally, the authors studied the effect of counter‐ions on the phase separation of lssDNA. They found no phase separation observed in the TE buffer (10 × 10^−3^ m Tris, 1 × 10^−3^ m ethylenediaminetetraacetic acid (EDTA), pH = 8) without Mg^2+^, which is because the charge on the phosphate skeleton is not shielded, and the DNA exhibits a state of electrostatic stability. For lssDNA containing poly A of ≈1000 bases in length, the phase transition caused by hydrophobic interaction occurs at ≈75 °C, 17.5 × 10^−3^ m Mg^2+^. When the Mg^2+^ concentration is increased to 100 × 10^−3^ m, the cloud point temperature reduces to 40 °C. The counter‐ion was changed from Mg^2+^ to Ca^2+^ (calcium chloride, CaCl_2_), and a similar phase transition was observed (Figure 2D). Note that large alkaline earth elements (such as barium chloride, barium chloride, ≤100 × 10^−3^ m) do not cause heat‐induced phase separation behavior of lssDNA, while the transition metal (zinc chloride, manganese chloride) can cause phase separation of lssDNA at room temperature (≥20 × 10^−3^ m).

We can conclude from the above work that, when the surface charge of DNA is screened by the presence of counter‐ions, the RCA‐produced lssDNA shows physical properties similar to thermoresponsive polymers. A certain temperature would induce hydrophilic and hydrophobic phase separation, distinct from the properties of oligonucleotides. Such phase separation behavior is related to the composition and the length of the NAs, the temperature, and the concentration of counter‐ions. Indeed, besides the hydrophobic interaction, some other factors also contribute to the self‐driving condensation of lssDNA, such as the nonspecific hydrogen‐bonding and purine–purine *π*–*π* stacking. The formation process of nanoparticles by lssDNA can be inferred as the following. The lssDNA can be well dispersed in water due to the electrostatic repulsion of phosphate backbone. A certain concentration of counterions, especially Mg^2+^, can screen the surface charge of lssDNA. The aromatic structures of lssDNA are hydrophobic, showing the tendency of aggregation and resulting in two situations. If lssDNA is super long, the phase separation of lssDNA will be observed without heat treatment; or, if lssDNA is of medium length (≈1000 base), it needs to be heated to cause phase separation.

### 2.1 DNA Nanomaterials Stabilized by Hydrophobic Interaction for Chemotherapy

Chemotherapy has become the indispensable cancer‐treatment method nowadays; however, this method shows serious drawbacks as anticancer drugs would nonspecifically attack both cancer cells and nonlesional normal cells. Therefore, tumor‐targeted and microenvironment‐responsive chemotherapeutics have attracted a lot of research interest.^[^ ^48^ ^]^ Functional nucleic acids (FNAs) have been widely applied to realize targeted drug delivery and release by its specific recognition ability. Recently, the pure DNA nanostructures, like DNA origami, become famous as the delivery system for its precise and smart controls. At the same time, they still face many challenges hindering its biomedical application, such as the unsatisfactory biostability for in vivo application, the tedious fabrication strategy, and the high expense of a large number of oligonucleotides.^[^ ^6^, ^17^, ^18^, ^49^ ^]^ The low‐stability in the serum‐containing medium is the foremost problem, the primary solutions to which includes reducing the number of nick sites, improving compaction density of DNA, and external encapsulation protection.

**Figure 3:**
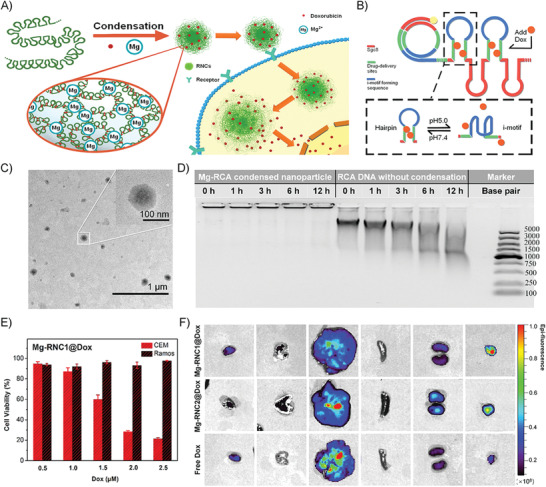
A) Illustration of the phase separation of Mg^2+^‐stabilized lssDNA nanoparticles for targeted Dox‐delivery. B) Illustration of the design of Mg‐RNC. The blue part will fold up at pH 5 to release Dox in the green part. The red part is sgc8 aptamer for targeting CEM cells. C) TEM imaging of Mg‐RNC with sizes of about 100 nm. D) Biostability of Mg‐RNC comparing with the free lssDNA. The “time” in the inset refers to the incubation time in serum. E) The cytotoxicity of Mg‐RNC1@Dox nanoparticles on targeted CEM cells and nontargeted Ramos cells. F) The distribution of Mg‐RNC1@Dox, Mg‐RNC2@Dox (without targeted aptamer sequences), and free Dox in the main organs after intravenous injection. From left to right are the hearts, lungs, livers, spleens, kidneys, and tumors. The pseudocolor indicates the fluorescence intensity of Dox. All panels are reproduced with permission.^[^ ^21^ ^]^ Copyright 2018. American Chemical Society.

Our group employed the RCA method as mentioned above to fabricate targeted chemotherapy‐based nanostructures.^[^ ^21^ ^]^ First, a carefully designed template was replicated through an RCA reaction to yield a large quantity of lssDNAs with periodic sequences, and each repeat is comprised of an aptamer sequence with the ability to target cancer cells and a hairpin sequence for doxorubicin (Dox)‐loading and pH‐responsive release (**Figure** **3**A,B). After that, a certain concentration of Mg^2+^ could effectively cause the firm condensation of the lssDNA due to the hydrophobic interaction, forming nanoparticles with a small size of ≈100 nm (Mg‐RNC). Besides, the Mg‐RNC could remain intact and stable after removing the excess Mg^2+^ through desalting. The Mg/P ratios (molar ratio of Mg^2+^ to DNA phosphate groups) play an important role in the formation of Mg‐RNC. As the Mg/P ratio increased from 0 to 200, the *R*_g_/*R*_h_ (*R*_g_: radius of gyration, *R*_h_: hydrodynamic radius) ratio decreased from about 1.5 to 0.77, which were monitored by laser light scattering (LLS). This phenomenon indicates that lssDNA has changed from a random coil conformation to a solid spherical structure. However, hardly any obvious conformation change could be observed if short oligonucleotides were mixed with excessive Mg^2+^, which indicates that the high molecular weight is of great significance to the Mg‐RNC formation.

Transmission electron microscopy (TEM) images of Mg‐RNC nanoparticles showed that the nanostructures are like “fried eggs” with relatively dense internal and irregular perimeters (Figure 3C). The stability of the drug delivery system in serum is of great significance for in vivo experiment. Compared with the pure RCA product, Mg‐RNC nanoparticles showed no significant degradation after a 12 h incubation in serum medium (Figure 3D). The aptamer (sgc8) targeting the receptor protein tyrosine kinase 7 (PTK7) on the cell membrane was integrated into the RCA product. These receptors are overexpressed in the human leukemic cell line (CEM) while low expressed in human lymphoma cell line (Ramos). The Dox‐loaded, targeting‐capable nanoparticles (Mg‐RCN1@Dox) were incubated with CEM and Ramos cells, respectively. The results showed that Mg‐RCN1@Dox only exhibited significant cytotoxicity to CEM, indicating that the prepared nanoparticles did show the selectively targeted cytotoxic effects (Figure 3E). Due to the high stability of Mg‐RCN nanoparticle, it can be used for targeted drug delivery in vivo. Different groups of samples were injected intravenously into tumor‐bearing mice. After 24 h of in vivo distribution, the mice were sacrificed, and the heart, liver, spleen, lung, and kidney were collected; and the fluorescence distribution of Dox was observed. The results showed that Mg‐RCN nanoparticles accumulate more concentrated in the tumor than free Dox, and the aptamer sequence improved the in vivo accumulation of nanoparticles at the tumor site (Figure 3F).

In a word, the counter‐ion Mg^2+^ can drive lssDNAs rather than short oligonucleotides from the random coil conformation to the compact solid spherical structure. DNA is a polyelectrolyte, the compaction of which could be restricted by charge‐repulsions. When the surface charge of DNA is fully screened by Mg^2+^, the intramolecular interactions (hydrophobic interaction in the majority) were enhanced due to the high‐molecular‐weight of lssDNA, and phase separation will take place to condense DNAs to form nanostructures, which are stable enough for in vivo chemotherapeutic applications. In summary, multifunctional lssDNA for targeted chemotherapy can be produced by RCA reaction, and the stable self‐delivery nanomedicines with superior biocompatibility could be further fabricated by the simple addition of Mg^2+^. Overall, this new strategy of exploiting the RCA technique and Mg^2+^ condensation, can fabricate nanoparticles with a nontoxic composition through a simple fabrication process and provides an efficient way to preserve and promote DNA functions, which shows the great potential for broad applications in the biomedical field.

### 2.2 DNA Nanomaterials Stabilized by Hydrophobic Interaction for Gene‐Therapy

RNA interference (RNAi) has proven to be an effective treatment strategy. However, the biggest hurdle that hinders RNAi therapy is the inefficient delivery. Short‐chain length with a rigid structure makes double‐stranded siRNA more difficult to form stable complexes with cationic transfection agents compared with the long plasmid DNA.^[^ ^50^, ^51^ ^]^ Moreover, these unstable complexes are more enzyme sensitive, giving rise to the early release of siRNA, which results in low cellular uptake, low serum stability, and even nonspecific immune responses. In addition, excessive dosage of cationic transfection agents can also pose many biosafety problems. siRNA polymerization has been proven to be a very promising strategy in enhancing the efficiency of siRNA delivery by changing the low‐charge and rigid properties of single siRNAs. Comparing with the methods of direct siRNA linkage, herein, we developed a novel method exploiting the RCA product to facilitate the high degree polymerization of siRNA and the subsequent formation of the robust complex with transfection agent poly(ethylenimine) (PEI).^[^ ^52^ ^]^

**Figure 4:**
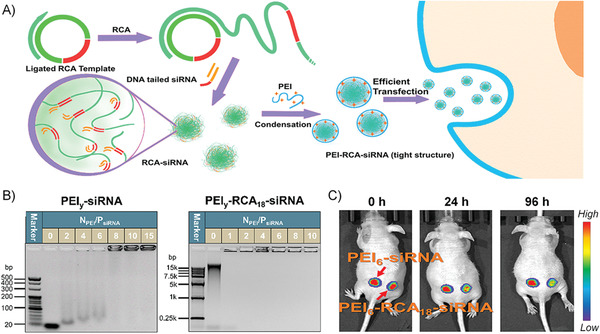
A) Illustration of the design principle: the RCA product acts as a cocarrier for siRNA delivery. B) Agarose gel electrophoresis analysis of PEI/siRNA and PEI/RCA‐siRNA polyplexes. C) The in vivo RNAi test by the intratumoral injection of PEI/siRNA and PEI/RCA‐siRNA polyplexes. All panels are reproduced with permission.^[^ ^52^ ^]^ Copyright 2019. American Chemical Society.

In this work, we carefully investigated and elucidated the role of the RCA lssDNA as a cocarrier material for siRNA delivery. The enzymatically synthesized lssDNA shows polymer‐like properties; therefore, its interaction with PEI is highly dependent on its molecular weight (MW). Since the MW and functionality of the DNA can be easily tailored according to the application of interest, which makes it surpass the other synthetic polymers. Through a simple sequence optimization, siRNAs could be efficiently hybridized to the RCA cocarrier, and the hybrid could be more efficiently complexed by PEI (**Figure** **4**A). We revealed that the length of the binding site was vital to efficient hybridization. The siRNA with an 18‐base DNA tail (DNA_18_–siRNA) was optimized to show a 100% graft efficiency, while neither DNA_12_–siRNA nor DNA_6_–siRNA showed satisfactory hybridization efficiency. Therefore, with the efficient hybridization, hundreds of siRNAs could be grafted to a single RCA chain, which will be the most efficient way among all the methods based on the siRNA “polymerization” strategy. The hybrid of siRNAs and the RCA cocarrier could be complexed by PEI more efficiently. Different from siRNA, the RCA product with a large spatial charge density and high molecular flexibility was proved to show stronger affinity to PEI, in which the sufficient complexation with PEI was achieved when the charge was completely neutralized. We found that the interaction between DNA and PEI is MW‐dependent, and the higher MW of RCA product brought the better stability of the polyplexes. The chain flexibility was decreased due to the introduction of more double‐stranded sites to RCA cocarriers during hybridization. However, the RCA‐siRNA hybrid system exhibited similar PEI complexation performance as RCA products. The loose complexation of PEI/siRNA is challenged by circulating nuclease degradation, renal clearance, and the reticuloendothelial system uptake. Although the excessive use of PEI can improve the stability of PEI/siRNA polyplexes, it will induce severe cytotoxicity at the same time. Starting from the *N*_PEI_/*P*_siRNA_ ratio of 2 (approximately at the charge neutralization point), the complexation structure of PEI/RCA‐siRNA could be well preserved in the biological environment, which ensured the further success of the polyplexes in cellular and in vivo applications (Figure 4B). Also, PEI/RCA‐siRNA polyplexes showed efficient cell uptake and proper siRNA release.

Finally, based on all these good properties, the best RNAi efficiency was determined to be 80% produced by PEI/RCA‐siRNA, which was better than the commercially available lipofectamine under the optimized conditions (≈60%). PEI/RCA‐siRNA polyplex was selected for the in vivo transfection investigation, which exhibited an in vivo gene suppression efficiency of 50% (Figure 4C). On the contrary, the PEI/siRNA complexes did not cause a significant decrease in the in vivo luciferase expression. In summary, free of any chemical processes, the very biocompatible RCA cocarrier could hybridize with hundreds of siRNAs, which could be sufficiently complexed by a reduced amount of PEI, resulting in a polyplex with low cytotoxicity and improved RNAi efficiency both in vitro and in vivo. Therefore, the potentials of the RCA cocarrier on improving siRNA delivery have been carefully proved, making this strategy promising for RNAi applications in the future.

### 2.3 DNA–AgNC Composite Nanomaterials Stabilized by Hydrophobic Interaction for Cell imaging and Intracellular Detection

With the excellent physical properties of good water solubility, convenient preparation, and high fluorescence, oligonucleotide‐stabilized silver clusters (AgNCs) have gained increasing attention in the biomedical field. Despite their rapid development, the low photo‐stability is still the major shortcoming of most oligonucleotide‐stabilized AgNCs. Especially for the AgNCs with the ability to emit red light, their emitting light would turn from red to green gradually within 24 h.^[^ ^53^, ^54^ ^]^ Therefore, our group tried to protect AgNCs with long and condensed RCA products to improve their stability for biological applications.^[^ ^55^ ^]^

**Figure 5:**
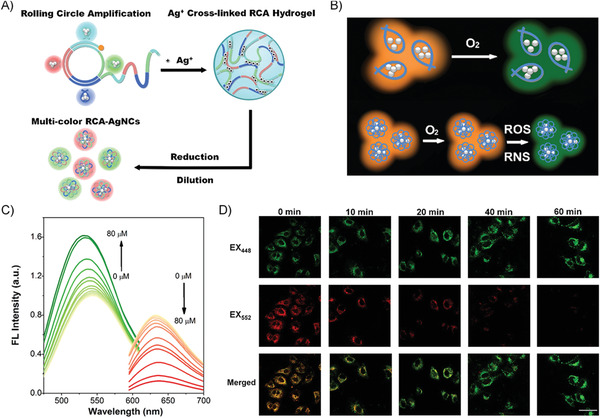
A) Illustration of the preparation of RCA‐AgNCs. B) Illustration of RCA‐AgNCs with higher photostability for detecting ROS/RNS. C) Titration curves of the RCA‐AgNCs with an increase in •OH concentration; the green emissions were excited at 440 nm and the red emissions were excited at 560 nm. D) Using the RCA‐AgNCs as a ratiometric fluorescent probe to monitor the dynamic levels of ROS/RNS in lipopolysaccharide‐treated A549 cells at various incubation times. All panels are reproduced with permission.^[^ ^55^ ^]^ Copyright 2018, American Chemical Society.

First, cytosine‐rich RCA products were synthesized, which were crosslinked by Ag^+^ to form DNA hydrogels. After reduction, the prepared fluorescent RCA‐stabilized AgNCs were carefully characterized (**Figure** **5**A). The (dynamic light scattering) DLS analysis showed that the diameter of the RCA–AgNC complex was about 230 nm, and the diameters of AgNCs were determined to be ≈2 nm characterized by TEM. Therefore, the nanocomposites of RCA–AgNC complexes are AgNCs encapsulated within the condensed RCA sequences, in which the hydrophobic interaction of RCA products played a considerable role to enhance the stability of AgNCs. In comparison with AgNCs stabilized by oligonucleotide, RCA‐stabilized AgNCs showed greatly enhanced photo‐ and thermostability and declined toxicity. As for photostability, the red emission of oligonucleotide–AgNCs decreased 80% in the ambient environment after 5 days preservation from light, while the RCA–AgNCs showed superior photo‐stability that there was only a little decline in red emission intensity even after 30 days under the same condition. Upon 10 h exposure to sunlight, the intensity of the red emissions of the oligonucleotide–AgNCs and RCA–AgNCs decreased by 70% and 15%, respectively. For the thermostability, when incubated at 37 °C for 60 min, the RCA‐AgNC remained ≈90% of its original fluorescence, while the oligonucleotide–AgNCs lost ≈80% of its fluorescence.

The selective detection of reactive oxygen or nitrogen species (ROS/RNSs) would be benefited from the superior photostability of RCA–AgNCs, as the common physical factors that cause emission quench could be ignored except for the ROS/RNSs (Figure 5B). The reversible response of AgNCs to redox was improved due to the protection by RCA products. Therefore, the RCA–AgNCs with comparable green or red emission were fabricated by rational DNA sequences design to realize multicolor cell imaging and ROS/RNSs ratiometric detection. The ratios of emission integrations (*R* = *I*_green_/*I*_red_) between the ranges of 500–560 nm (from the green‐emission of AgNCs) and 610–700 nm (from the red‐emission of AgNCs) in the absence and the presence of ROS/RNS, were calculated to evaluate the sensitivity of RCA–AgNCs in response to certain ROS/RNS species (Figure 5C). The ratio significantly increased over 10 times for the presence of •OH, slightly increased by ≈3 times for single oxygen (^1^O_2_), and negligibly increased for the other ROS/RNS, such as alkylperoxyl radical (ROO•), hypochlorite (ClO^−^), peroxynitrite (ONOO^−^), and H_2_O_2_. All the results certified that RCA–AgNCs showed the best selectivity for the detection of •OH (the most reactive form of oxygen), with a low limit of detection (LOD) reaching 58 × 10^−9^ m. In the meantime, RCA–AgNCs could be successfully applied to monitor the dynamic levels of ROS/RNS in lipopolysaccharides treated human lung cancer (A549) cells (Figure 5D). These results demonstrated that the RCA–AgNC with outstanding fluorescence property and application‐oriented advantages is a promising nanomaterial for biomedical applications in multicolor cell imaging and intracellular sensing.

## 3 Hydrophobic Interaction Based on DNA Amphiphiles

Another strategy to stabilize DNAs by hydrophobic interactions is to conjugate DNA with hydrophobic organic molecules, like dyes, drugs, polymers, or dendrimers, to produce DNA–organic hybrid amphiphiles, which will self‐assemble into many higher‐order structures in aqueous solutions, such as micelles, tubes, and vesicles of various shapes. These novel DNA amphiphilic materials show many distinctive properties due to the incorporation of the hydrophobic block.^[^ ^36^, ^37^, ^38^, ^39^, ^40^, ^41^, ^56^ ^]^ 1) As a result of the self‐assembly driven by hydrophobic–hydrophilic phase separation, the conjugated DNAs are densely packed at the outer shell of the resultant nanostructures, which will enhance the biological stability and cellular penetration capability of DNAs, and finally make them more suitable for intracellular and in vivo applications.^[^ ^57^ ^]^ 2) Meanwhile, the hydrophobic domains of the self‐assembled nanostructures can load hydrophobic drugs or organic dyes. Except for increasing the their solubility in water, the DNAs packed at the outer shell will enhance drug capacity in targeted and stimuli‐responsive delivery.^[^ ^50^ ^]^ 3) Besides, the lengths and sizes of the hydrophilic DNA and hydrophobic part will substantially affect the self‐assembly structures, as DNAs can go through conformational switches in response to environmental stimulus, DNA–organic hybrid amphiphiles can behave more intelligently in biomedical applications.^[^ ^58^, ^59^ ^]^ On the basis of the above features, DNA–organic hybrid amphiphiles have been applied in a variety of fields, including biosensors, gene regulation, and drug delivery. In the following sections, we will discuss the synthesis of DNA amphiphiles and summarize their recent applications.

### 3.1 The Synthesis of DNA–Organic Hybrid Amphiphiles

DNA–organic hybrid amphiphiles are synthesized by the conjugation of hydrophilic short‐strand DNA and hydrophobic blocks through the covalent bonds. Since DNA and most hydrophobic molecules are highly immiscible, the corresponding conjugation reactions become relatively difficult. The interesting properties and application potentials of DNA amphiphiles have driven researchers to develop different methods to promote conjugation reactions.^[^ ^56^ ^]^ 1) Hydrophobic molecules are directly conjugated to the terminal of DNAs through a common coupling reaction using either solid‐phase or liquid‐phase reaction.^[^ ^59^ ^]^ 2) A hydrophobic polymer is directly polymerized from the initiator which is attached to the DNA end.^[^ ^60^, ^61^, ^62^, ^63^ ^]^ (3) A polymerizable monomer is attached to a DNA unit and DNA‐grafted amphiphilic polymer is synthesized as a result of the polymerization.^[^ ^64^ ^]^

Several kinds of coupling reactions are commonly used to synthesize DNA–organic hybrid amphiphiles. For example, amidation between amine and carboxyl group, disulfide bond, Michael addition reaction, and copper‐catalyzed (or copper‐free) cycloaddition reaction.^[^ ^41^, ^56^ ^]^ Amide bonds are unstable to the alkaline environment, and the disulfide bonds can be broken when encountering reducing substances. The chemical bonds formed by the latter two reactions are relatively stable to the environment and therefore are more broadly used. Some strategies have been developed to improve the yields of the heterogeneous reactions between hydrophobic molecules and DNAs. For instance, Herrmann's group reported a method for obtaining efficient DNA amphiphiles in liquid‐phase reaction, where the positively charged surfactant can bring DNAs to the organic solvent, thereby greatly improving the reaction yield.^[^ ^65^ ^]^ Alternatively, the coupling reactions were conducted in solvents that can dissolve both DNA and hydrophobic molecules to a comparable extent, such as dimethylformamide (DMF), dimethylsulfoxide (DMSO), or a mixed solution of the first two with water.^[^ ^50^, ^66^ ^]^ Besides, the solid‐phase reaction can also improve the yields of DNA amphiphiles, where the solid support can bring DNAs into the organic solvent system under vigorous stirring;^[^ ^67^, ^68^ ^]^ Also, there are some attempts to prepare DNA amphiphiles by the polymerization of monomer‐conjugated DNA or directly run polymerization from the initiator‐conjugated DNA.^[^ ^61^, ^64^ ^]^ In addition, the purification of DNA amphiphiles is also a key issue, and several purification methods have been developed according to the characteristics of the DNA amphiphiles. These methods include 1) the separation is proceeded by the differences in molecular weights, such as dialysis, ultrafiltration, and electrophoresis gel,^[^ ^69^ ^]^ 2) reversed‐phase chromatography exerts separation by the polar differences,^[^ ^70^, ^71^ ^]^ 3) size exclusion chromatography exerts separation by the differences in molecular sizes,^[^ ^72^, ^73^, ^74^, ^75^ ^]^ and 4) anion exchange chromatography exerts separation by the charge density differences.^[^ ^76^ ^]^

**Figure 6:**
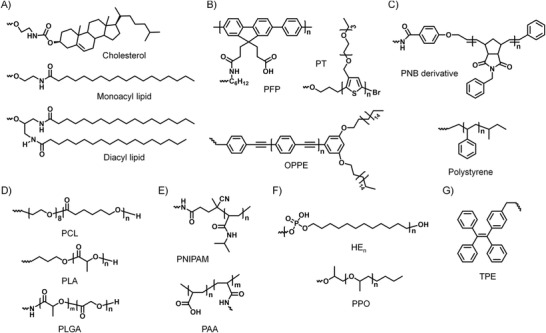
The structural formula of commonly used hydrophobic moieties of DNA amphiphiles. A) Lipids: cholesterol, monoacyl lipid, and diacyl lipid. B) Molecules with *π*‐conjugated systems: poly[fluorene‐phenylene] derivative (PFP), polythiophene derivative (PT), and oligo‐*p*‐phenyleneethynylene derivative (PPE). C) Strongly hydrophobic polymers: polynorbornene (PNB) derivative grafted with aromatic rings and polystyrene (PS). D) Biodegradable polymers: polylactic acid (PLA), polycaprolactone (PCL), and polylactic acid‐glycolic acid (PLGA). E) Stimulus‐responsive polymers: temperature‐responsive poly(*N*‐isopropylacrylamide) (PNIMAP) and pH‐responsive polyacrylic acid (PAA). F) Other hydrophobic molecules: polyphosphorylated hexaethylene (HE*_n_*), polypropylene oxide (PPO). G) Typical AIE molecule: tetraphenylethylene (TPE).

The most commonly used hydrophobic moieties (**Figure** **6**A–G) of DNA amphiphiles can be roughly divided into the following categories: 1) lipids (such as fatty chains, cholesterol, and their analogs),^[^ ^77^, ^78^, ^79^, ^80^ ^]^ 2) *π*‐conjugated molecules (including hydrophobic fluorescent dyes and conjugated polymers),^[^ ^76^, ^81^, ^82^ ^]^ 3) strongly hydrophobic polymers (polystyrene, polynorbornene (PNB) derivatives grafted with aromatic rings),^[^ ^74^, ^75^ ^]^ 4) biodegradable polymers ((polylactic acid (PLA), polycaprolactone (PCL), and polylactic acid–glycolic acid (PLGA)),^[^ ^66^, ^83^, ^84^, ^85^, ^86^ ^]^ 5) stimulus‐responsive polymers ((temperature‐responsive poly(*N*‐isopropylacrylamide) (PNIMAP),^[^ ^64^ ^]^ pH‐responsive polyacrylic acid (PAA)),^[^ ^87^ ^]^ 6) some other hydrophobic molecules ((poly phosphorylated hexaethylene (HE*_n_*),^[^ ^88^ ^]^ polypropylene oxide (PPO)),^[^ ^89^, ^90^, ^91^ ^]^ and 7) aggregation‐induced emission (AIE)‐based molecules.^[^ ^92^, ^93^ ^]^ These hydrophobic moieties are chemically conjugated to DNAs (with different sequence lengths) through covalent bonds, producing various DNA amphiphiles.

### 3.2 DNA Amphiphile‐Based Biosensor

**Figure 7:**
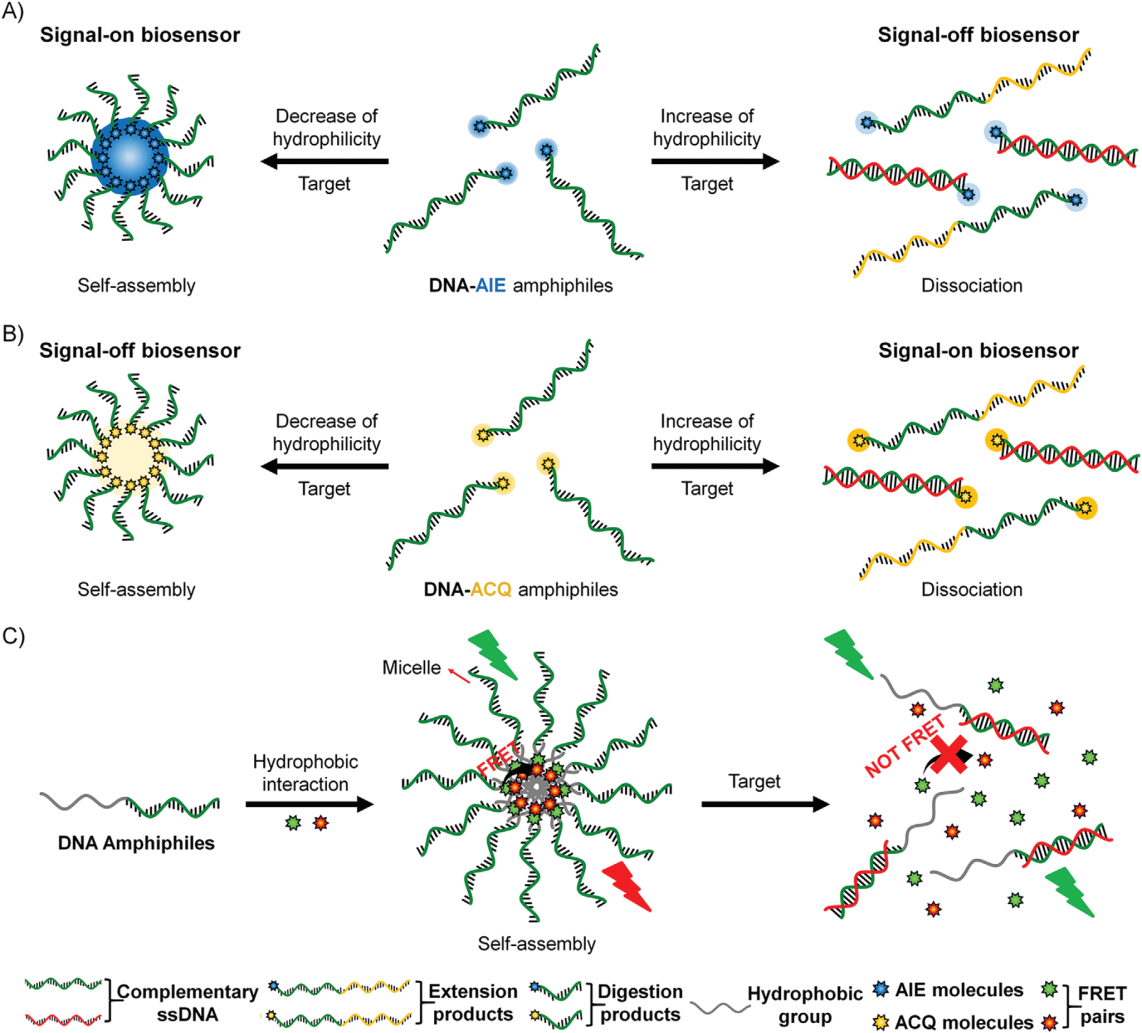
Several strategies for constructing fluorescence biosensors based on DNA amphiphiles. Schematic illustration of the strategy of signal‐on/off biosensors based on A) DNA‐AIE amphiphiles and B) DNA‐ACQ amphiphiles. C) Schematic representation of fluorescence biosensors based on the self‐assembly of DNA amphiphiles through hydrophobic interactions.

DNA‐based biosensors play a significant role in many fields, especially in disease diagnosis, environmental monitoring, and food analysis. Sensitivity is crucial for the development of biosensors, which relies on the sensitive and specific recognition of targets and the subsequently triggered signal transduction.^[^ ^94^ ^]^ The specific Watson–Crick base‐pairing between DNA strands guarantees that DNA‐based biosensors can detect the complementary DNA with high sensitivity and fidelity. Besides, developed by SELEX, aptamers are short, single‐stranded DNA or RNA that can selectively bind to specific targets, which broadens the targets of DNA‐based biosensors from DNA/RNA to micromolecules (such as ATP and ions), large biomolecules (proteins), and even whole organisms (cells and tissues).^[^ ^95^, ^96^ ^]^ In pursuit of higher sensitivity, numerous signal transduction approaches have been developed for DNA‐based biosensors, including fluorescence, electrochemistry, colorimetry, and surface plasmon resonance, and so forth. Among them, the fluorescence‐based detection method is mostly used. Molecular beacon is a DNA hairpin structure that is widely used as fluorescent probes. Initially, the fluorescence is quenched by the dual‐modification DNA ends with fluorophore and quencher, which will be restored when the molecular beacon hybridizes to a complementary target sequence. Similarly, many DNA‐based fluorescence probes are developed by DNA‐conformational‐switch controlled fluorescence changes.^[^ ^97^ ^]^ In addition to this design strategy, DNA amphiphiles bring new insights into the design of DNA‐based fluorescence probes.^[^ ^98^ ^]^ On one side, DNA amphiphiles generally self‐assemble into nanostructures through hydrophobic interactions. When the DNA block recognizes targets by chain hybridizations or conformational changes, the amphiphilicity of the self‐assembly system will be altered to result in further aggregation or dissociation of the previous nanostructures. On the other side, in addition to the dependence on fluorophore/quencher pair, many hydrophobic dyes showing aggregation‐state‐sensitive fluorescence properties can be applied to the design of DNA amphiphile based biosensors. For instance, when the hydrophobic dyes with aggregation‐induced emission (AIE) characteristic, the target‐induced aggregation will turn on the DNA amphiphile biosensor (**Figure** **7**A); while for the hydrophobic dyes showing the property of aggregation‐caused quenching (ACQ), the target‐induced aggregation will result in fluorescence turn off (Figure 7B). Moreover, hydrophobic dyes can be encapsulated into the hydrophobic core of the DNA amphiphile self‐assembly to realize biosensing and detection (Figure 7C). In this section, we focus on the discussions of fluorescence biosensors based on DNA amphiphiles, for which the signal transduction is through the target‐triggered amphiphilicity changes.

#### 3.2.1 Fluorescence Biosensors Based on DNA Amphiphiles

Most organic fluorescent materials exhibit ACQ effect that their fluorescence will be quenched at high concentrations or in the aggregated state, which limits their application as probes since they cannot be used at high concentrations.^[^ ^99^ ^]^ However, these materials with the ACQ characteristics can be used for the construction of signal‐on or signal‐off biosensors that based on the change of amphiphilicity of the conjugates. Since Tang and co‐workers discovered the hexaphenylsiloles (HPS) with phenyl rotors structure that emits strong fluorescence in the aggregated state in the year 2001, this kind of aggregation‐induced emitters (AIEgens) with the characteristics of aggregation‐induced emission has provided an alternative candidate for the fluorescence transducer.^[^ ^100^ ^]^ We classify these biosensors into signal‐on and signal‐off by the photophysical property of the coupled organic material.

##### Signal‐On

**Figure 8:**
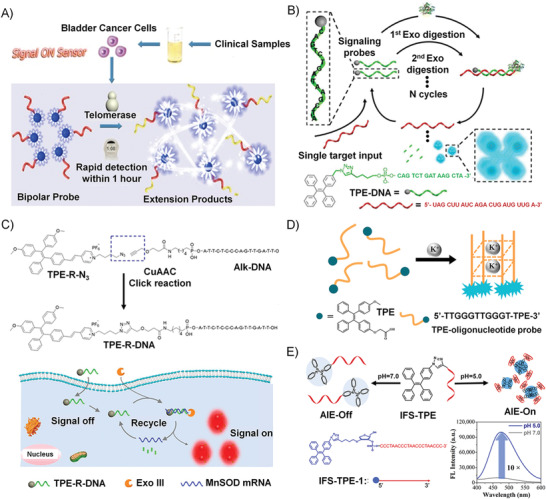
A) Schematic illustration of the strategy of telomerase activity detection based on the amphipathic bipolar CP‐c‐DNA probe through changing the hydrophilicity of the probe. Reproduced with permission.^[^ ^101^ ^]^ Copyright 2015, American Chemical Society. B) Schematic representation of TPE‐DNA probe to detect miR‐21 with the assistance of EXO III. Reproduced with permission.^[^ ^105^ ^]^ Copyright 2015, American Chemical Society. C) Upper panel: Synthetic route of TPE‐R‐DNA. Lower panel: Schematic representation of TPE‐R‐DNA probe to detect MnSOD mRNA. Reproduced with permission.^[^ ^106^ ^]^ Copyright 2018, American Chemical Society. D) Schematic illustration of TPE‐DNA probe for K^+^ detection. Reproduced with permission.^[^ ^107^ ^]^ Copyright 2017, Elsevier. E) The working scheme of IFS‐TPE conjugates in response to pH changes, and the fluorescence intensity of AIE increased when the pH was switched from 7.0 to 5.0. Reproduced with permission.^[^ ^93^ ^]^ Copyright 2018, Royal Society of Chemistry.

Conjugated polymer (CP) is a typical fluorescent material that has been widely used for the design of biosensors due to its light‐harvesting and energy transfer capability. For example, Xia's group designed a conjugated polymer‐DNA (CP‐c‐DNA) bipolar beacon. The amphiphilic nature of CP‐c‐DNA drives the formation of a micelle, and the fluorescence of CPs is quenched due to the ACQ effect. The elongation of the DNA length will impair the micelle stability and induces the collapse of micelles, resulting in a signal‐on sensing system. Therefore, this system was applied to the detection of telomerase activity by using a telomerase substrate sequence as the DNA block (**Figure** **8**A).^[^ ^101^ ^]^ Telomerase is a common biomarker for most immortal cell lines and primary human tumors, which adds multiple TTAGGG strands to the end of telomere. In the presence of telomerase, the telomerase substrate sequences conjugated to CP‐c‐DNA micelle were catalyzed by telomerase to extend to longer chains, as a consequence, the hydrophilicity of the DNA part increased and led to the collapse of the micelles, resulting in the recovery of CP fluorescence. The LOD of this signal‐on biosensor was estimated to be 4 bladder cancer cells per microliter (µL), which is comparable to the PCR method. In comparison with PCR and other DNA amplification methods, this method is accurate and simple by avoiding numerous artifacts and sophisticated optimizations, which was successfully applied in both mimic systems and real urine samples, offering a cost‐effective, simple, and noninvasive method for bladder cancer diagnosis.

The AIEgens found by Tang's group show high brightness and photobleaching resistance in the water, which are more suitable for the design of signal‐on biosensors. Several DNA–AIEgen conjugates have been developed for applications in optical sensing and imaging.^[^ ^99^ ^]^ By coupling the hydrophilic DNA segment, the DNA–AIE conjugates display satisfactory water‐solubility that ensures the low fluorescence background in bioassays. Liu and Tang et al. reported the first “turn‐on” probe based on a DNA–AIE conjugate for specific NA detection in 2013.^[^ ^102^ ^]^ They created a DNA–AIE probe (TPE‐DNA) consisting of a typical AIEgen (tetraphenylethene, TPE) and a 20‐mer ssDNA sequence. Principally, the AIE effect is mainly caused by the restriction of intramolecular motions (RIM).^[^ ^103^ ^]^ Therefore, when the DNA–AIE probe hybridized with its complementary DNA strand, the rigid conformation and hydrophobic stacking interactions of the double helix structure caused the RIM process of TPE, resulting in a 3‐fold enhancement in the fluorescence intensity. The LOD of this DNA‐TPE probe was calculated to be 0.3 × 10^−6^ m of complementary DNA. To pursue higher signal‐to‐noise ratio (SNR) and sensitivity, Liu and Tang et al. developed a two‐armed TPE–DNA conjugate (TPE–2DNA), in which two oligonucleotides were conjugated to one TPE moiety.^[^ ^104^ ^]^ TPE–2DNA probe showed a lower fluorescence background in the absence of target due to its enhanced solubility in water. Meanwhile, as a result of the presence of two arms, it displayed a stronger fluorescence after target recognition.

MicroRNAs (miRNAs) are a kind of noncoding ssRNAs, and the dysregulations of miRNAs expressions are closely allied to many diseases. Xia's group devised a water‐soluble amphiphilic TPE–DNA probe to detect miR‐21.^[^ ^105^ ^]^ The TPE–DNA probe contained a TPE group and a particular DNA sequence that can hybridize with miR‐21, as shown in Figure 8B. The resultant DNA–RNA duplex would be recognized by exonuclease III (EXO III), and therefore the DNA part was digested to release the target miR‐21 and the residual TPE units. The released miR‐21 target could be recognized by another TPE‐DNA probe, resulting in a cyclic process and amplified detection signal. The residual TPE units aggregated in water and lit up due to the AIE effect. Later, Xia and co‐workers designed a TPE‐R‐DNA probe based on the same EXO III‐assisted strategy for the detection of manganese superoxide dismutase (MnSOD) mRNA (Figure 8C).^[^ ^106^ ^]^ They demonstrated the hydrophobic residue TPE‐R‐AT aggregated in water and emitted strong fluorescence. The LOD of the TPE‐R‐DNA was as low as 0.6 × 10^−12^ m in vitro. What is more, TPE‐R‐DNA could detect MnSOD mRNA in cancer tissues, proving its application capability in bioimaging.

Functional ssDNAs can not only hybridize with their complementary strands but also can bind to diverse target molecules (such as metal ions, organic dyes, amino acids, and enzymes) by folding into distinct secondary or tertiary structures.^[^ ^108^ ^]^ For instance, DNAs can form double‐ or triple‐stranded structures, inter‐ or intramolecular G‐quadruplexes or i‐motif structures, which have been widely utilized in the conformation‐switch‐based DNA biosensors.^[^ ^109^ ^]^ The combination of the AIEgens with the specific recognition ability of the functional ssDNAs will develop many new capable biosensors. Guanine‐rich DNA sequences can fold into a quadruplex structure known as G‐quadruplex by the stacking of four Hoogsteen‐paired guanines.^[^ ^110^ ^]^ DNA quadruplexes can be further stabilized by the addition of alkali‐metal cations (such as Na^+^ and K^+^) through electrostatic interactions. The monitoring of K^+^ is vital and urgent because of their unique relationship with various diseases. Tan and Zhang et al. designed an AIE‐based DNA probe (TPE‐DNA) for K^+^ detection, in which a water‐soluble TPE derivative was chemically conjugated with a guanine‐rich short DNA via the amide bond.^[^ ^107^ ^]^ As seen in Figure 8D, in the presence of K^+^, the K^+^‐induced parallel G‐quadruplex structure brought four TPE molecules into proximity, and hindered the intramolecular motions of TPE, resulting in a fluorescence‐signal enhancement. Both of the in vitro and cellular results proved that this AIE probe showed a ∼10‐fold higher sensitivity over other G‐quadruplex probes.

As illustrated in Figure 8E, our group synthesized a new DNA‐based hybrid fluorescent probe containing an i‐motif forming sequence (IFS) and monofunctionalized TPE, which showed a distinct pH‐responsive AIE effect.^[^ ^93^ ^]^ We demonstrated that the conformational switch of the IFS block triggered by a change of external pH could well control the hydrophobicity of the IFS‐TPE hybrid, which was applied in pH‐responsive biosensing. Cytosine‐rich DNA sequences can undergo a conformational transition from a random coil to a folded i‐motif structure under acidic conditions.^[^ ^111^ ^]^ In a neutral environment, the IFS‐TPE probe existed as a random‐coil conformation, while the IFS block folded into the i‐motif structure at acidic pH and induced the AIE effect. A 10‐time AIE enhancement was observed by turning the pH from 7.0 to 5.0. There were two reasons for the outstanding pH‐responsive AIE effect. One was due to the hydrophobic stacking interaction. TPE molecules were stacked on the i‐motif quadruplex structure when the IFS sequence folded in response to a decrease in pH, which restricted the internal rotations of TPE and initiated the AIE effect. The other reason is that the solubility of the hybrid molecule in water will be reduced due to IFS folding, which facilitated the aggregation of the TPE part and initiated the AIE effect. The TEM image indicated that, at an acidic condition, IFS‐TPE would form spherical aggregates with diameters of 150–200 nm. Moreover, this turn‐on fluorescent probe owned great resistance to oxidation and held the potential for pH monitoring in the complex intracellular environment. As a result, the signal‐on strategy based on the pH‐induced AIE effect described in this work will be significant for the development of novel DNA‐based probes in the future.

##### Signal‐Off

Besides the signal‐on fluorescent probe, a collection of the signal‐off biosensor was designed for particular biosensing. The above‐mentioned conjugated polymer (CP)–DNA conjugates developed by Xia's group can also be used as a signal‐off biosensor for Hg^2+^ detecting.^[^ ^101^ ^]^ The CP–DNA conjugates were in the dispersed state with the initial fluorescence of CPs, while in the presence of Hg^2+^, the hydrophobic parts of CP aggregated together by the formation of T–Hg^2+^–T complexes. Also, this sensing strategy can be extended to detect various other analytes (such as other metal ions or proteins), by just replacing the recognition elements of the CP–DNA with other functional DNA sequences such as aptamers.

#### 3.2.2 DNA Biosensors Based on the Self‐Assembly of DNA Amphiphiles through Hydrophobic Interactions

The predictable and programmable features make DNAs become the ideal scaffolds for the design of DNA‐based biosensors.^[^ ^112^, ^113^, ^114^, ^115^ ^]^ Recently, quite a few DNA biosensors based on the self‐assembly of DNA amphiphiles are developed. Roelfes et al. proposed a novel design of DNA–lipid conjugates, and the folding of the DNA parts into G‐quadruplex structures drives the formation of micelle aggregates.^[^ ^116^ ^]^ Upon the addition of the complementary DNA strand, the hybrid double helix structure unfolded G‐quadruplexes that made the micelle disassemble, resulting in cargo release. A well‐known Förster resonance energy transfer (FRET) pair (two dyes) was simultaneously encapsulated in this micelle, which showed the donor emission as a result of the efficient FRET. When the micelles disassembled in the presence of complementary DNA, the two dyes were released, resulting in decreased FRET efficiency. Furthermore, this system was applied to the detection of adenosine 5’‐triphosphate (ATP). A DNA hairpin containing the ATP‐binding DNA aptamer was designed, which was comprised of an ATP‐binding domain and a responsive domain. In the absence of ATP, the responsive domain was locked in the stem region of the hairpin; and upon binding of ATP, the hairpin structure was rearranged to expose the responsive domain, which could hybridize with the DNA–lipid micelles and resulted in cargo release. The presented design convinced the applicable versatility of this strategy for the detection of different kinds of targets or stimuli.

**Figure 9:**
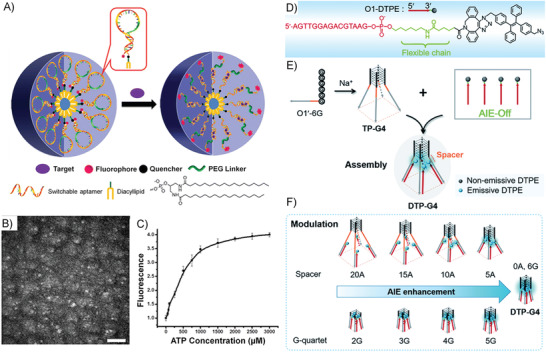
A) Working principle of the switchable aptamer micelle flares formed by DNA‐diacyllipid conjugates. B) TEM image of the switchable aptamer micelle flares after negative staining by 2% aqueous uranyl acetate (scale bar: 200 nm). C) The in vitro responses of switchable aptamer micelle flares to ATP target. The panels (A)–(C) are adapted with permission.^[^ ^117^ ^]^ Copyright 2013, American Chemical Society. D) Schematic illustration of the molecular structure of O1‐DTPE, E) using the G‐quadruplex structure as the molecular scaffold for AIEgen self‐assembly, and F) the accurate control of AIE effect by the G‐quadruplex structure. The panels (D)–(F) are adapted with permission.^[^ ^118^ ^]^ Copyright 2018, Royal Society of Chemistry.

As shown in **Figure** **9**A, Tan and Yang et al. designed a switchable aptamer micelle flare (SAMF) formed by the self‐assembly of DNA–diacyllipid conjugates (the DNA part is a hairpin structure with the sequence of ATP aptamer locked in the stem region) to detect and monitor ATP molecules in live cells.^[^ ^117^ ^]^ The strong hydrophobic interaction made the DNA–diacyllipid conjugates form a uniform spherical nanostructure as clarified by the TEM image (with a diameter of 28 nm as shown in Figure 9B), displaying quenched fluorescence due to the ACQ effect. Upon the binding of target ATP, the hairpin DNA rearranged its conformation and destabilized the self‐assembled nanostructure to make fluorescence restored (Figure 9C). As the diacyllipids in SAMF are similar to the cell membranes, SAMFs could be efficiently uptake by live cells compared with other DNA probes. Therefore, SAMFs with properties of cell permeability and the controllability at nanoscale show application potentials in bioanalysis, disease diagnosis, and drug delivery.

Lei et al. made a molecular scaffold based on a tetrapod DNA quadruplex (TP‐G4) for self‐assembly and precise control of the AIE‐effect‐based fluorescence signal transduction.^[^ ^118^ ^]^ As shown in Figure 9D, the scaffold TP‐G4 was formed by four O1ʹ‐6G strands that can provide a confined space for the regulation of the AIE effect. A DNA–AIEgen conjugate, O1‐DTPE, was synthesized by “copper‐free” click coupling, which was water‐soluble and exhibited low fluorescence in water. When O1‐DTPE was self‐assembled with TP‐G4, the fluorescence of DTPE became a 10.4‐fold higher than the background. The significant AIE effect was induced by the aggregation of AIEgens refined by the G‐quadruplex structure (Figure 9E). Besides, as depicted in Figure 9F, the AIE effect could be precisely regulated by the distance between the G‐quadruplex core and the AIEgens and by altering the quartet number of the G‐quadruplex. Furthermore, similar self‐assembly and AIE regulation system was developed by using i‐motif as the clawed scaffold, showed a structure‐dependent light response to both tetra‐ and bimolecular i‐motif quadruplex structures. These results demonstrated that DNA scaffolds could efficiently regulate the AIE effect, which could form a universal molecular tool for the design of new biosensing strategies.

### 3.3 DNA Amphiphile‐Based Biomedicine

In medicine, agents with pharmacological activity are called drugs. The efficacy of drugs is not only related to their chemical formula but also depends on their dosage form and routes of administration, which can be evaluated by pharmacokinetics, the study of the time course of drug absorption, distribution, metabolism, and excretion. Chemotherapeutic drugs and NA drugs play a pivotal role in cancer treatment.^[^ ^48^, ^119^ ^]^ Commonly used chemotherapy drugs include Dox,^[^ ^91^ ^]^ paclitaxel (PTX),^[^ ^57^ ^]^ and camptothecin (CPT),^[^ ^120^ ^]^ and NA drugs include ASOs,^[^ ^70^ ^]^ siRNAs,^[^ ^50^ ^]^ DNAzymes,^[^ ^64^ ^]^ aptamers,^[^ ^74^ ^]^ and immune adjuvant CpG.^[^ ^79^ ^]^ Generally, both drugs cannot be used directly due to their disadvantages in biomedical applications, which will affect their in vivo pharmacological activities. Therefore, materials science and engineering has been more and more involved in drug design and formulation. Nanodrug delivery system (NDDS) based on drugs and other materials was designed to solve the problems of chemotherapy drugs, such as poor water solubility, severe toxicity and side effects, rapid renal excretion, weak biological stability, low cell absorption efficiency, and nonspecific immune inflammation.^[^ ^48^ ^]^ In addition, NDDSs could facilitate the accumulation of drugs in solid tumor sites due to the well‐known enhanced permeability and retention (EPR) effect, resulting in targeted drug delivery. NA drugs also suffer from instability in biological environments and poor cellular uptake due to their high negative charge.^[^ ^6^ ^]^ Self‐assembly and conjugation strategies have been developed to enhance the biostability and cellular penetration properties of NA drugs.^[^ ^41^, ^56^ ^]^

Recently, DNA amphiphiles attract great research attention due to its capability in improving the pharmacokinetics and pharmacodynamic properties of both chemotherapy drugs and NA drugs.^[^ ^50^, ^57^, ^68^, ^71^, ^83^ ^]^ Driving by hydrophobic interactions, DNA amphiphilic molecules can self‐assemble into nanostructures, such as micelles or vesicles in water. For micellar nanostructure, the hydrophobic core of micelles can carry insoluble drugs, such as Dox,^[^ ^91^ ^]^ PTX,^[^ ^57^, ^121^ ^]^ and CPT.^[^ ^120^ ^]^ Vesicle shows a relatively complicated nanostructure, in which the hydrophilic core can be used to transport some hydrophilic drugs, and the hydrophobic bilayer can carry hydrophobic drugs.^[^ ^50^ ^]^ As for the DNA part, the densely packed DNAs in the aggregate of DNA amphiphiles show a “cluster” effect, which become more resistant to nuclease degradation and more capable in cell and tissue penetration compared with the free DNAs. Meanwhile, the DNA functions can be well reserved and even enhanced under the dense‐pack‐state. Therefore, the NA drugs can be used as the DNA block of the amphiphile or introduced through chain hybridization with the DNA block. More than the dual enhancements to the two kinds of drugs, the size, morphology, and surface properties of the nanostructures of DNA amphiphiles can be well‐tuned by adjusting the chemical structures of the hydrophobic block and the hydrophilic DNA block, which will significantly affect the pharmacological activity and kinetics of drugs. In this section, we summary the design principle of NDDSs based on DNA amphiphiles and exemplify their applications.

#### 3.3.1 Design Principle of Delivery System Based on DNA Amphiphiles

**Figure 10:**
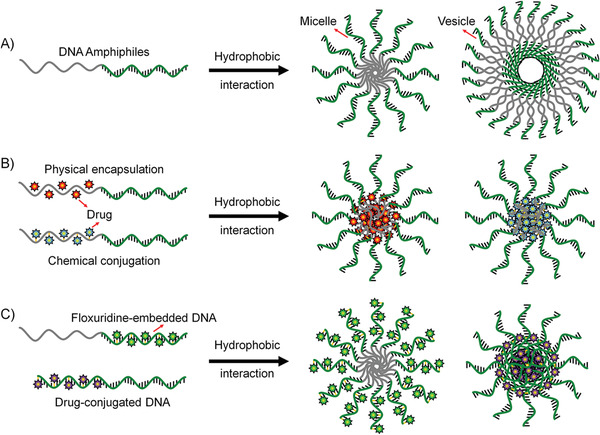
Several strategies of constructing DNA amphiphiles to prove the in vivo performance of therapeutic NAs. A) DNA amphiphile can self‐assemble into 3D nanostructures such as micelle and vesicle, which can enhance the biostability and intracellular delivery of NAs. B) The micelles formed from DNA amphiphiles contain a hydrophobic environment that can accommodate the lipophilic molecules through the physical encapsulation. Alternatively, the drug molecules are chemically conjugated to the hydrophobic moiety of the DNA amphiphile. C) Floxuridine, a typical anticancer nucleoside drug, is embedded into the DNA sequence by replacing the T‐base due to their structural similarity. Benzyl bromide‐modified drugs can react with phosphorothioate groups to achieve the direct chemical conjugation of drugs to DNA without the need for additional drug carriers.

The following points need to be considered when designing NDDs based on DNA amphiphiles for practical applications: 1) the NDDs must well protect drugs from early leakage and efficiently deliver drugs to the target site; 2) as for the design of cancer drugs, the drugs also need to be taken up by target cells and reach subcellular organelles to work; 3) in addition, it is better that the NDDSs can respond to tumor microenvironments and specifically release drugs. The recently developed DNA amphiphile‐based NDDSs can be roughly classified into the following categories (**Figure** **10**).

##### 3D Structure Improving the Performance of Nucleic Acid

NA drugs, such as ASO, aptamer, siRNA, DNAzyme, and CpG DNA, have been proven to possess good pharmacological activity in preclinical and clinical applications. However, the inherent properties of NA drugs, such as low stability in biological environments, poor cell penetration, and undesired immune response, limit their direct application in clinical practice.^[^ ^6^ ^]^ Many gene delivery vectors (such as cationic liposomes, viral vectors, and inorganic materials) have been developed to improve the biological properties of NA drugs. Moreover, recently, the development of NA nanotechnology has opened up new directions for the delivery of NAs. Nucleic acid nanostructures, such as DNA origami, framework NA, and spherical nucleic acid (SNA), have made important contributions to improving the bioavailability of NA drugs in in vivo applications.^[^ ^17^, ^18^, ^19^ ^]^ Among them, nanostructures, such as micelles or vesicles, self‐assembled from DNA amphiphiles, which are chemical conjugation of pharmacologically active NAs and hydrophobic molecules, have attracted more and more attentions in NA drug delivery (Figure 10A). These micelles and vesicles have densely packed NAs on their surfaces, exhibiting “spherical nucleic acid” properties, which show extraordinary biomedical activity compared with free nucleic acids.^[^ ^25^ ^]^ 1) the densely packed NAs are resistant to nucleases by preventing the accessibility; 2) the self‐assembled nanostructures become more permeable to cells through the interactions with the receptors on the cell surface, which can mediate polyanion transcytosis; 3) It is also reported that nanostructures with densely packed NAs on the surface can cross the blood‐brain barrier (BBB) through receptor‐mediated transcytosis to achieve diagnosis and therapy of central system diseases.

##### Drug‐Loaded DNA Amphiphile Micelle

Only when the drug reaches the target tissue or cell can it play an ideal pharmacological effect, especially for chemotherapy drugs. Naked chemotherapeutic drugs reach around the body only through concentration‐dependent diffusion and cannot distinguish diseased cells from normal cells. Therefore, direct intravenous injection of naked chemotherapeutics shows risks in producing great harms such as undesired side effects and systemic toxicity. The use of NDDs can alleviate this problem to some extent. Many drug carriers have been applied for targeted delivery of chemotherapeutics, such as liposomes, polymeric micelles, inorganic materials, and metal–organic complexes. In particular, amphiphiles can self‐assemble into 3D nanostructures through hydrophobic interaction in aqueous solutions, and these nanostructures include hydrophobic domains and hydrophilic domains. These two domains can simultaneously accommodate drugs with different properties to achieve synergetic diagnosis and treatment (Figure 10B). The inner core provides a hydrophobic environment for hydrophobic drugs, including but not limited to Dox, PTX, and CPT.^[^ ^57^, ^91^, ^120^ ^]^ For NDDS fabrication, these lipophilic drugs and DNA amphiphiles are first dissolved in good solvents; later, by solvent exchange, the two will form drug‐loaded DNA amphiphile micelles, which can significantly enhance the bioavailability of naked drugs due to the better targeted‐cell‐delivery capabilities.

##### DNA–Amphiphile–Drug Conjugates

Instead of direct encapsulating drugs into the DNA amphiphile micelles, the conjugation of drugs to DNA amphiphiles provides another approach to realize efficient drug delivery, which shows the following characteristics (Figure 10B): 1) drug can be loaded in a well‐defined stoichiometric ratio; 2) it shows better stability than physical encapsulation, as the conjugation through covalent bonds will prevent premature drug release. For fabrication, Drug molecules are conjugated to the hydrophobic moieties of DNA amphiphiles, which can form drug‐conjugated micelles due to hydrophobic interactions in aqueous solutions for eventual application in targeted delivery of chemotherapeutics.

##### Floxuridine‐Embedded DNA Amphiphiles

Floxuridine is a nucleoside analog, which is used as an antitumor drug.^[^ ^70^, ^122^ ^]^ Due to its structural similarity to thymine (T) nucleoside, fluorouridine can replace T in the NA sequence without affecting the base‐pairing capability. Therefore, floxuridine can be embedded in the NA sequence by replacing T during DNA solid‐phase synthesis (Figure 10C). The resulting floxuridine‐embedded DNA is then chemically conjugated with a hydrophobic moiety to form a floxuridine‐embedded DNA amphiphile. This special amphiphile can form 3D micelles through hydrophobic interactions in aqueous solution, which helps it efficiently penetrate cells to kill cancer cells.

##### DNA–Drug Amphiphiles

Despite DNA amphiphiles have proven to be safe and nontoxic to a large extent. However, the safety of hydrophobic moieties introduced into DNA amphiphiles remains to be further confirmed. Taking all‐DNA carriers can avoid this problem because DNA is an endogenous material showing good biocompatibility.^117^ During the solid‐phase synthesis, phosphorothioate groups are used to replace phosphodiester groups, which can react with a benzyl bromide group to form a chemical conjugation. Therefore, benzyl bromide‐modified hydrophobic drugs can be grafted into the phosphodiester sites on the DNA backbone (Figure 10C). The resulting DNA^PS^–drug conjugates show obvious amphiphilicity and can self‐assemble into 3D micelle structure through hydrophobic interaction in aqueous solution. This drug delivery strategy by the conjugating drugs to the NA backbone can well control the drug‐NA ratio and show low safety risks.

##### Intelligent Design Based on DNA Amphiphiles

To sum up, the DNA amphiphile‐based NDDSs show the following advantages: 1) DNA amphiphile‐based NDDS shows precise and consistent drug‐loading capability, which can be achieved through chemical conjugation between chemotherapy drugs and DNA amphiphiles. 2) The densely packed structure of NAs on the surface of NDDS micelles formed by the self‐assembly of DNA amphiphiles can delay the nuclease degradation; an additional shell of polyethylene glycol (PEG) can also be designed to enhance the stability of DNA micelles during blood circulation and avoid rapid clearance by the liver. 3) Taking advantage of DNA nanotechnology, intelligent NDDSs can be designed to improve the pharmacological activity of drugs. For instance, large self‐assembly structures can be fabricated to conceal the targeting ligands, which will accumulate at the tumor site due to the EPR effect. At the tumor site, in response to tumor microenvironments, the large structure can be disassembled into small nanoparticles, exposing the active ligands on the surface to mediate the internalization of the nanoparticles.

#### 3.3.2 Instance of Applications

Since the design principle of NDDSs based on DNA amphiphiles has been elucidated in the above part. In this section, we will introduce the instances of their applications in the field of biomedicine.

##### Improving the Performance of Functional NAs through Hydrophobic Interaction

1) ASOs: ASOs are short ssDNA, of which the length is 8–50 nucleotides. The function of ASOs was first observed by Zamecnik, and he proved that virus replication could be restrained through downregulating protein expression with the existence of the specific oligonucleotides.^[^ ^123^ ^]^ After that, the mechanism beneath the function of ASOs has been revealed. After the hybridization of ASO with its corresponding mRNA through Watson–Crick base pairing, the formation of the DNA–RNA complex would induce RNase H assisted degradation. The degradation of the targeted mRNA interrupts RNA processing, resulting in the inhibition of protein expression. There are some alternative mechanisms for the modulation of gene expression via ASOs, such as the steric blockade of RNA binding proteins, and splicing modulation.^[^ ^124^ ^]^ Although ASOs have great clinic application potentials and several ASOs drugs have already been approved by Food and Drug Administration (FDA) such as fomivirsen for cytomegalovirus retinitis,^[^ ^125^ ^]^ mipomersen for homozygous familial hypercholesterolemia (HoFH),^[^ ^126^ ^]^ eteplirsen for Duchenne muscular dystrophy (DMD),^[^ ^127^ ^]^ and nusinersen for spinal muscular atrophy (CMA),^[^ ^128^ ^]^ the existing challenges including autoimmunity, off‐target effect, sensitive to nuclease, and low cell membrane across efficacy, still limit its further development.^[^ ^129^ ^]^ Some methods based on DNA–hydrophobic conjugates have been proposed by researchers to overcome these challenges.

**Figure 11:**
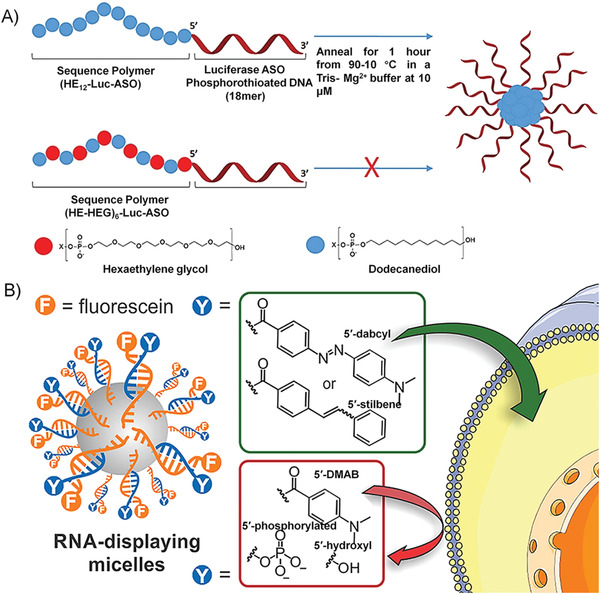
A) Schematic representation of HE_12_‐Luc‐ASO and (HE‐HEG)_6_‐Luc‐ASO with different micelles forming capability for the delivery of antiluciferase ASO. Reproduced with permission.^[^ ^51^ ^]^ Copyright 2015, Royal Society of Chemistry. B) Schematic representation of the RNA‐polymer amphiphile for siRNA delivery; some chemical modifications, 5ʹ‐dabcyl, 5ʹ‐stilbene, 5ʹ‐DMAB, 5ʹ‐phosphorylated, and 5ʹ‐hydroxyl, on the outer siRNAs were investigated, and only dabcyl and stibenen could help to enhance the transfection efficiency of siRNA. Reproduced with permission.^[^ ^75^ ^]^ Copyright 2018, American Chemical Society.

ASOs can hardly diffuse across the cell membrane freely because of the intrinsic negative charged characteristics, which severely limits its gene regulation function.^[^ ^12^ ^]^ Therefore, vectors are needed to realize gene delivery. Although modified virus vectors are widely applied to enhance the delivery efficiency, the unsolved safety problem of immunogenicity and high cost cannot be ignored. Some synthetic cationic nonviral vectors have been investigated to decline immunogenicity and cost for improving transfection performance.^[^ ^16^ ^]^ Among these vector compounds, PEI holds excellent performance of combining DNA with its positively charged amino moieties. However, the cytotoxicity of PEI becomes the fundamental concern for its clinical viability. Sleiman and co‐workers have designed and constructed a novel ASO–polymer conjugate that maintains high gene knockdown efficiency while less PEI vectors needed, resulting in low cytotoxicity.^[^ ^51^ ^]^ They fabricated three samples to investigate their cytotoxicity and gene knockdown capability. The first one was phosphorothioate ASO against firefly luciferase (Luc‐ASO) as the control. The other two were the same ASOs conjugated to the twelve dodecane units (HE_12_‐Luc‐ASO) and the polymer (with alternating dodecane units and hexamethylene glycol, (HE‐HEG)_6_‐Luc‐ASO), respectively. Owing to the hydrophobicity of dodecane units, HE_12_‐Luc‐ASO could self‐assemble to form spherical micelles via a simple annealing process. By contrast, the presence of hydrophilic hexamethylene glycol in (HE‐HEG)_6_‐Luc‐ASO decreased the hydrophobicity of the polymer chain and led to the inhibition of spherical micelles formation (**Figure** **11**A). For the subsequent application, PEI was introduced to form ASO‐conjugate:PEI complexes. They observed that Luc‐ASO: PEI and (HE‐HEG)_6_‐Luc‐ASO:PEI complexes showed poor gene silencing capability. The gene silencing activity could be significantly enhanced and only observed for HE_12_‐Luc‐ASO:PEI, even with the PEI concentration as low as 1 µg mL^−1^. The compact micelles structure driven by hydrophobic interaction could efficiently reduce the usage of PEI for transfection and gene silencing, which provides a safe and efficient strategy for gene therapy.

By scavenger receptor (SR)‐mediated internalization, spherical nucleic acids (SNAs) that show the high efficiency in gene regulation without the need for auxiliary transfection reagents have turned to be more and more attractive.^[^ ^130^ ^]^ Zhang and co‐workers proposed a novel approach to graft ASOs onto the hydrophobic PCL block, an FDA approved clinical application material, to form a biodegradable amphiphilic DNA brush block copolymer (DBBC) with the ability to self‐assemble into SNAs.^[^ ^83^ ^]^ DBBCs could form uniform monodisperse micelles due to the strong hydrophobic interaction. Compared with conventional linear DNA block copolymer (LDBC), the micelles of DBBC could be functionalized with more NAs on the surface. The high density of surface NAs endowed it with a high melting temperature, about 3.5 °C higher than that of LDBC‐SNAs. Therefore, when the ASO against enhanced green fluorescent protein (EGFP) was introduced to SNA structures by the chain hybridization, the DBBC‐SNAs showed better cell uptake efficiency and more effective gene knockdown efficiency than the LDBC‐SNA (54% vs 37% knockdown efficiency). The biodegradable perspective of DNA‐PCL in the physiology environment reduced adverse effects caused by the inappropriate accumulation, and this DBBC design could be broadened to other biodegradable hydrophobic materials, such as PLA and PLGA. The superiorities of DBBC‐SNAs, such as transfection‐reagent‐free internalization and effective gene regulation efficiency, promise this kind of DNA nanomaterials the great potentials in clinical application.

As discussed above, the formation of SNAs has been considered as an efficient strategy for gene delivery.^[^ ^25^ ^]^ Generally, inorganic nanoparticles, like AuNPs, are widely used to construct the SNA structures, which, however, will result in the risk of cytotoxicity caused by inorganic nanoparticles.^[^ ^131^ ^]^ To solve this problem, Gianneschi and co‐workers fabricated organic SNA material for mRNA regulation, which showed comparable cell uptake efficiency to Au‐SNA.^[^ ^73^ ^]^ This novel material was constructed by conjugating hydrophobic polynorbonyl and hydrophilic NA through the amidation coupling of carboxylic acid and amine on each segment. The novel DNA amphiphile self‐assembled into micelles driven by hydrophobic interaction. The highly dense NA corona at the outside of micelles still maintained its biofunctions. The micelles were characterized by TEM and DLS with a uniform size of about 10 nm in radius. For practical application, the locked nucleic acid (LNA), showing enhanced biostability and hybridization stability than the normal NAs, was conjugated with the polymer to synthesize the LNA‐polymer amphiphile (LPA). The cellular uptake was verified by fluorescence‐activated cell sorting (FACS), which revealed that LPA showed cell uptake efficiency 10 times higher than that of single‐stranded LNA. Consequently, by introducing the antisurvivin LNA, the resultant LPA materials showed a very efficient mRNA regulation capability. The mechanism of the uptake of LPA within the cell was investigated; as methyl‐*β*‐cyclodextrin could significantly decrease the internalization of LPA, Class A SR‐mediated endocytosis pathway was confirmed.^[^ ^132^ ^]^ Rather than utilizing inorganic nanoparticles, organic SNAs exploited synthetic polymer and hydrophobic interaction to construct SNA structures, which made the gene‐delivery system more biocompatible for clinical applications. Moreover, the diversified structures and the tunable properties of synthetic polymers can provide broad possibilities for the DNA amphiphiles.

Although some traditional delivery systems for therapeutic NAs have been explored, there still exist hurdles, such as the off‐target problem, hampering its clinical translation.^[^ ^133^, ^134^ ^]^ To solve this limitation, Sleiman's group proposed an idea to design a stimuli‐responsive SNA that could precisely inhibit a specific gene in target cells.^[^ ^135^ ^]^ The SNA was assembled by two “pillar” like DNA–polymer conjugates, partially hybridized by a “bridge” like DNA, on which the ASO “cargo” against luciferase could be further hybridized. The responsive process could be initiated by the overexpressed miRNA in target cells, which subsequently triggered the release of the ASO for mRNA regulation. This SNA system delivered genes only in response to the presence of miRNA biomarkers, which could improve the performance of ASO therapy.

2) siRNA: As a new type of gene therapy, RNA interference (RNAi) has attracted broad attention. Numerous laboratory and clinical studies have been carried out and showed great application promise for diseases caused by gene mutation as well as the abnormal gene expression.^[^ ^136^ ^]^ siRNAs are short dsRNA, with 21–23 base pairs in length, which are delivered in duplex for stability. After entering the cell, siRNA is recognized and loaded onto argonaut‐2 protein (AGO2) to form the AGO2‐RISC, the RNA‐induced silencing complex. The sense strand of a siRNA is hydrolyzed, and the residual antisense strand can guide the cleavage of specific mRNA sequences to inhibit the expression of the corresponding protein. The key challenges for siRNA therapy come from the delivery and off‐target issues, so the design and construction of stable, nontoxic, and in vivo effective delivery system for siRNA are urgently needed.^[^ ^137^ ^]^

Although some siRNA delivery applications have been carried out by inorganic SNAs,^[^ ^138^, ^139^, ^140^ ^]^ there still exist drawbacks, such as the nanoparticle‐determined chemical functionalization efficiency and the accumulation‐induced cytotoxicity.^[^ ^141^ ^]^ Gianneschi's group synthesized an RNA‐polymer amphiphile, which self‐assembled into organic SNAs with about 100 RNA strands on the surface.^[^ ^75^ ^]^ Moreover, the surface‐conjugated RNAs could be further hybridized with the complementary sequences to form siRNAs for gene silencing via RNAi pathway.^[^ ^142^ ^]^ To enhance its stability against RNase mediated degradation, the 2’‐fluoropyrimidine (2’‐F) moiety, a proven nuclease‐resistance reagent,^[^ ^143^ ^]^ was modified to the RNA strands; as a result, only little hydrolysis was observed even in the presence of a high concentration of RNase A (100 ng µL^−1^). For intracellular uptake, they found that the internalization of this RNA‐polymer SNA was not as efficient as that of the previously reported DNA‐polymer SNA, which was probably because the secondary structures of RNA induced unfavorable receptor recognition. However, they also found that some chemical moieties, such as dabcyl and stilbene, can enhance the transfection efficiency of the RNA‐polymer SNAs (Figure 11B), resulting in a silence efficiency up to 90% for the survivin mRNA. This platform provides a convenient approach for siRNA loading via chain hybridization, and the introduction of certain chemical moieties on SNA surfaces offers a simple and straightforward way to modulate cellular uptake capability of SNAs.

3) CpG: The phenomenon that gene could trigger immune responses was first found in 1984. The specific DNA extracted from Mycobacterium tuberculosis could activate nature killer (NK) cells and inhibit the proliferation of cancer cells.^[^ ^144^ ^]^ The mechanism of tumor regression caused by specific bacterial DNA later was proved owing to the existing unmethylated CpG sites within the sequences. The accumulation of CpG DNAs in antigen‐presenting cells (APCs) in lymphatics will bind with toll‐like receptor 9 (TLR 9) and initiate the immunity pathway, thereafter, promote the proliferation of cytotoxic CD 8^+^ cells. Now, the synthetic oligonucleotides with CpG sites have been widely investigated and utilized as efficient vaccine adjuvants.^[^ ^10^ ^]^ Nevertheless, many challenges remain to be overcome, such as safety, stability, and efficient delivery to the lymph node (LN) where the immune process takes place.

**Figure 12:**
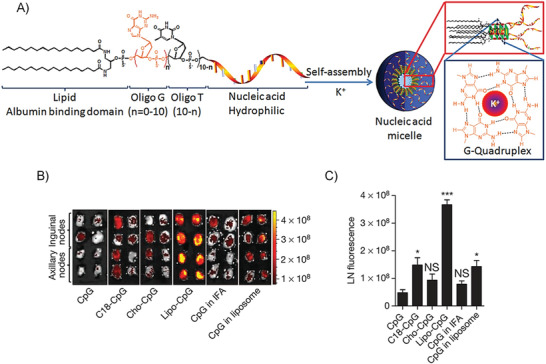
A) Schematic representation of the micelles self‐assembled from CpG‐Diacyl lipid amphiphiles. B) Fluorescence imaging of draining lymph nodes from C57BL/6 mice which were injected with different samples. C) the quantification of CpG accumulations. All panels are reproduced with permission.^[^ ^79^ ^]^ Copyright 2014, Nature Publishing Group.

To break the barriers of systemic toxicity and the sophisticated design, Irvine and co‐workers synthesized a DNA amphiphile comprised of the albumin‐binding lipid and the CpG DNA to enhance the delivery to LN via “albumin hitchhiking” process (**Figure** **12**A).^[^ ^79^ ^]^ Different amphiphiles with different lipophilic structures, such as cholesterol‐CpGs, monoacyl lipid‐CpGs and diacyl lipid‐CpGs, were synthesized to optimize the albumin‐binding capability. Only the CpG conjugates comprised of diacyl lipids could form liposomes driven by hydrophobic interaction and showed efficient albumin binding, which led to 30‐fold increases in T‐cell priming and enhanced antitumor efficacy while greatly reducing systemic toxicity (Figure 12B). Further, they proved that although the formation of micelle structure was critical for the effect of the CpG‐lipid amphiphiles, the micelles also had to disassemble into free amphiphile accompanied by interacting with albumin for taking effect. When poly‐G sequences were introduced between the diacyl lipid and CpG sequence to lock the amphiphiles in the micellar state by forming G‐quadruplex hydrogen bonding and block disassembly, the accumulation at LN was found decreased significantly. Therefore, moderate hydrophobic interaction plays an essential role in the effectiveness of this amphiphile system, which could ensure the dissociation of the micelle structure upon binding with albumin (Figure 12C). When combined with antigen, these CpG‐lipid adjuvants showed obvious tumor regression ability. Liu and co‐workers utilized this delivery system to examine the immune effects of different CpG sequences and proved the importance of LN‐ targeting for its vaccine adjuvant clinic application.^[^ ^11^ ^]^ This novel delivery design via “albumin hitchhiking” strategy could also be applied to other gene immunity therapeutics.

##### DNA Conjugates Work as Chemotherapeutic Drugs Carrier

Because of the cytotoxicity of chemotherapeutic drugs to healthy cells, the development of selective delivery to target cells becomes increasingly important. DNA‐*b*‐PPO block copolymer was synthesized by Herrmann as carriers to encapsulate Dox within the particle core by hydrophobic interaction.^[^ ^91^ ^]^ Through hybridization with folic acid linked complementary DNA, the targeting ability was realized owing to the high expression of the folate receptors on several kinds of cancer cells. The monodisperse particle of 10 nm with more folic acid ligands showed higher uptake efficiency and a better chemotherapeutic effect.

Precise delivery and release of drugs gain extensive attention nowadays. Zhang's group proposed a novel approach that could precisely control drug release via UV light activation by using the delivery system of DNA amphiphiles.^[^ ^120^ ^]^ They synthesized DNA amphiphiles by conjugating camptothecin (CPT) to DNA sequences that were modified with the photo‐cleavable linker, 2‐nitrobenzyl ether. Micelles of different morphologies, such as sphere and rod, could be easily formed in aqueous solution. The highly dense DNA‐corona could protect surface DNA from nuclease degradation, and only under the UV trigger, could the interior hydrophobic core release CPT and fulfill the chemotherapeutic function. If incorporated with two‐photon or NIR dye, this platform would gain the potential of precise spatiotemporal drug release.

**Figure 13:**
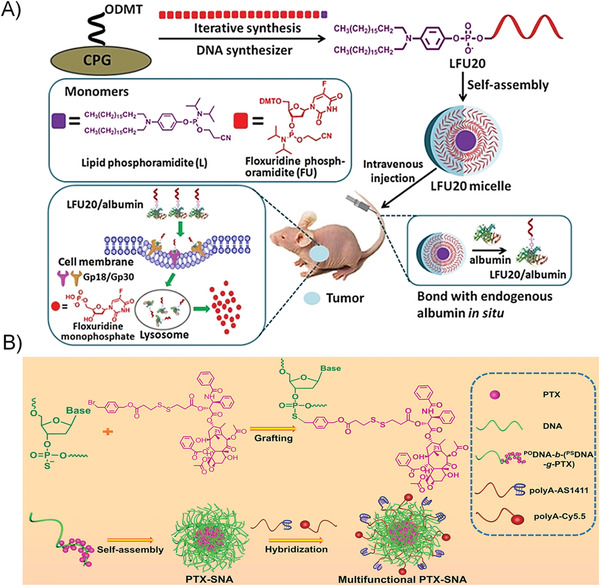
A) Schematic illustration of the synthesis of FU20‐lipid amphiphile and its enhanced systemic delivery by the “hitchhike” strategy. Reproduced with permission.^[^ ^122^ ^]^ Copyright 2018, Wiley‐VCH. B) Illustration of the synthesis of ^PO^DNA‐*b*‐(^PS^DNA‐*g*‐PTX) and its self‐assembled SNA for multifunctional biomedical applications. Reproduced with permission.^[^ ^121^ ^]^ Copyright 2019, Wiley‐VCH.

Not only DNA amphiphiles can form micelles and work as drug‐delivery vehicles, but some nucleobase analog drugs can also conjugate to hydrophobic molecules and enhance their biomedical function. Tan and co‐workers developed a lipid‐conjugated floxuridine homomeric oligonucleotide (FU20), a widely used therapeutic nucleobase analogue, the systemic delivery of which could be enhanced by “hitchhike” with albumin (**Figure** **13**A).^[^ ^122^ ^]^ Owing to the modification of diacyl lipid, the FU20‐lipid amphiphile self‐assembled into micelles and could noncovalently attach with albumin through the interaction of lipid and albumin hydrophobic core during blood circulation, which resulted in a sufficient tumor accumulation. After the endocytosis of cancer cells, FU20‐lipid/albumin complexes would be hydrolyzed by lysosome to release FU20 and inhibited cancer cell proliferation. This platform provides an idea of designing drug‐delivery systems for nucleoside/nucleobase analogue drugs.

##### Therapeutic DNA Conjugates Integrated with Chemotherapeutic Drugs for Synergistic Therapy

One of the main challenges that hinder the development of chemotherapeutic drugs in experimental and clinical practice is drug resistance.^[^ ^145^ ^]^ The degree of drug resistance mainly correlates with the expression level of the responsible proteins, so the incorporation with gene therapy provides a potential way to address the drug‐resistance issue of chemotherapy. Zhang's group constructed a chemogene SNA to realize the codelivery of therapeutic NAs as well as the chemotherapeutic drugs.^[^ ^57^ ^]^ One of the most widely used chemotherapy drugs, PTX, was attached to norbornenyl group via reductively cleavable disulfide linker and prepolymerized by ring‐opening metathesis polymerization. Through the “click” chemistry, the PTX grafted polymer was subsequently conjugated to a DNA strand, which was an ASO (G3139) targeting the antiapoptotic B‐cell lymphoma 2 (Bcl‐2) family proteins. With a degree of polymerization of 10, PTX_10_ provided enough hydrophobicity for the amphiphiles to form SNA micelles. The micelles with 15 nm diameter presented superior stability against DNase I and the high cell uptake efficiency. With the ability to load and release both PTX and ASO at the same time, this dual delivery design showed the inspiration for the clinic treatment of drug‐resistant cancers.

Other than using exogenous hydrophobic polymer to graft with PTX, Zhang and co‐workers designed an SNA material that was self‐assembled from the DNA strands modified with hydrophobic PTX (Figure 13B).^[^ ^121^ ^]^ The amphiphilic DNA molecule was composed of therapeutic Bcl‐2 ASO and PTX‐grafted phosphorothiolate DNA (^PO^DNA‐*b*‐(^PS^DNA‐*g*‐PTX)) conjugated together through the disulfide linkers. The SNAs were self‐assembled owing to the amphiphilicity, which could be subsequently functionalized with targeting aptamers and imaging dyes via hybridizations. After the aptamer‐assisted internalization, the structure of SNA would be broken through the cleavage of disulfide linkers by GSH and released free ASO and PTX. Treated by this SNA system, the expression of Bcl‐2 was downregulated by nearly 77% for drug‐resistant models both in vitro and in vivo, and the growth of tumor was substantially hindered by this synergistic treatment.

Besides the drugs attaching with DNA via a disulfide linker, Zhang's group proposed a novel drug conjugating approach to directly replace the thymine (T) on the DNA strand by nucleoside analog floxuridine (F).^[^ ^70^ ^]^ The F‐embedded Bcl‐2 ASO was conjugated to PEG‐*b*‐PCL through copper‐free click reaction. The forming micelles with controllable sizes regulated by PCL length would preferentially accumulate in tumors owing to the EPR effect. Though T was replaced by F, the knockdown efficiency of the ASO was still retained, which could efficiently reverse the drug‐resistance caused by Bcl‐2 protein. Meanwhile, the chemotherapeutic F would be released after the DNA strand was degraded by DNase. This synergistic chemo‐gene therapeutic system shows the obvious effect of reversing drug‐resistance, which could efficiently inhibit the growth of both orthotopic and subcutaneous drug‐resistant BEL‐7402 (human liver carcinoma cell line) tumors.

##### NIR‐II Emitting Organic SNA for Brain Tumor Imaging

**Figure 14:**
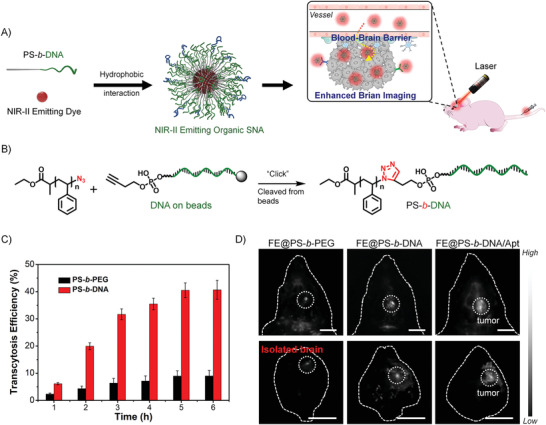
A) Scheme of the preparation of NIR‐II emitting organic SNAs and their application in brain tumor imaging. B) Scheme of PS‐*b*‐DNA synthesized by solid‐phase “click” reaction. C) Transcytosis efficiency for different samples over time. D) NIR‐II fluorescence imaging of the mouse heads and isolated brains by using different NIR‐II emitting materials under irradiation at 808 nm. All panels are reproduced with permission.^[^ ^68^ ^]^ Copyright 2020, Wiley‐VCH.

Brain tumor is one of the diseases that highly affect human health, whose visualization is critical for early diagnosis and imaging‐guided surgery. Compared with commonly used X‐ray computed tomography and magnetic resonance (MRI), fluorescence imaging is easier to operate and allows real‐time imaging during the operation. Among them, the second near‐infrared region (NIR‐II, 1000–1700 nm) fluorescence can provide a more promising method with deeper penetration depth and better resolution than NIR‐I fluorescence.^[^ ^146^ ^]^ However, the BBB hinders the imaging and diagnosis of brain tumors by using NIR‐II nanofluorophore.^[^ ^147^ ^]^ Therefore, our group developed a kind of organic SNA whose hydrophobic core can accommodate NIR‐II emitting fluorescent dyes. The resultant NIR‐II emitting organic SNA can effectively cross BBB and target brain tumors, thereby enhancing the diagnostic imaging of brain tumors (**Figure** **14**A).^[^ ^68^ ^]^

In order to prevent the early leakage of organic dyes in in vivo applications, polystyrene (PS) with high hydrophobicity was selected as the hydrophobic block to synthesize DNA block copolymer, PS‐*b*‐DNA. As shown in Figure 14B, PS‐*b*‐DNA was successfully synthesized by a solid‐phase “Click” reaction. After cleaved from beads, PS‐*b*‐DNA directly assembled into spherical micelles with the size of ≈20 nm in aqueous solution, which was determined by TEM and DLS. The densely packed DNAs on the surface of PS‐*b*‐DNA SNA showed hybridization ability comparable to free DNA. As for biostability, the in vivo half‐life of micellar DNA was determined to be ≈65 min, which was much larger than the that of free DNA (≈19 min), because dense DNA arrangements hinder the accessibility of nucleases.

Compared with the counterpart PS‐*b*‐PEG, PS‐*b*‐DNA SNA showed ≈3‐fold enhanced cell penetration ability, measured by FCM. Specific aptamers were introduced into the PS‐*b*‐DNA SNA with 10% of the total surface DNAs to obtain PS‐*b*‐DNA/Apt. The cell penetration ability of PS‐*b*‐DNA/Apt was determined to be 1.5‐fold stronger than that of PS‐*b*‐DNA SNA. Scavenger receptor (SR) was proved to be a key receptor for PS‐*b*‐DNA SNA penetrating cells through competitive inhibitor experiments. What's more, SR‐mediated transcytosis is a promising strategy for crossing BBB. Therefore, the ability of PS‐*b*‐SNA to cross BBB was evaluated, and the results indicated that PS‐*b*‐DNA showed 4.5‐fold higher traversing efficiency than that of PS‐*b*‐PEG (Figure 14C).

The NIR‐II emitting organic dye (FE) was encapsulated into the PS‐*b*‐DNA polymer matrix by using nanoprecipitation method to obtain FE@PS‐*b*‐DNA. The SNA with 25 wt% FE showed the brightest brightness and a fluorescence quantum yield of 9.6%. FE@PS‐*b*‐PEG and FE@PS‐*b*‐DNA/Apt were also prepared under the same conditions. Subsequently, in vivo brain tumor imaging was performed. At 24 h administration of different samples, FE@PS‐*b*‐DNA and FE@PS‐*b*‐DNA/Apt showed strong fluorescence signal at brain tumor sites compared with FE@PS‐*b*‐PEG. After imaging experiment, the isolated brains were collected. The enhanced accumulation of FE@PS‐*b*‐DNA was observed due to the ability of PS‐*b*‐DNA in BBB crossing; the targeted aptamer further enhanced the tumor penetrating ability of FE@PS‐*b*‐DNA/Apt. Therefore, compared with FE@PS‐*b*‐DNA, FE@ PS‐*b*‐DNA/Apt showed a 3.8‐fold signal enhancement (Figure 14D). Finally, the biocompatibility of FE@ PS‐*b*‐DNA has also been demonstrated, showing excellent biosafety. These results indicate that as a versatile polymer matrix, PS‐*b*‐DNA can facilitate functional organic dyes to cross BBB for the diagnosis and imaging of brain diseases.

## 4 Hydrophobic Interaction Based on Complex between DNA and Other Materials

In the above sections, the self‐assemblies of individual DNA and DNA amphiphiles driven by hydrophobic interactions for biomedical applications have been discussed. In addition to behaving as the driving force for self‐assembly, the hydrophobic interaction can also be applied to fabricate the composite materials between DNA and inorganic nanoparticles/organic molecules, which is also an important approach in the design of DNA‐based biomedical materials. Therefore, in this section, we will summarize the DNA‐containing composite materials due to the hydrophobic interactions.

### 4.1 Complex between DNA and Inorganic Nanoparticle

In the field of materials science, the superior properties of a variety of inorganic nanomaterials have been extensively investigated,^[^ ^148^ ^]^ for example, the superparamagnetism of magnetic nanoparticles, the optical properties of quantum dots, the surface plasmon resonance of AuNPs, and the upconversion emission of rare‐earth‐based nanoparticles.^[^ ^149^, ^150^, ^151^, ^152^ ^]^ In addition to these interesting properties, many inorganic nanomaterials show high cellular‐uptake capability and high tumor‐accumulation by the EPR effect. They are widely applied in biomedical applications, such as theranostics and biological detection.^[^ ^153^, ^154^, ^155^, ^156^ ^]^ As nanomaterials have large surface‐area‐to‐volume ratios,^[^ ^157^ ^]^ the complexation with functional biomolecules, including phospholipids, proteins, and nucleic acids, through surface adsorption has become a practical strategy to construct the biomedical materials.^[^ ^158^, ^159^, ^160^, ^161^ ^]^ In this section, we focus on discussing the use of hydrophobic interactions to form complexes between inorganic nanomaterials and NAs.

#### 4.1.1 GO–DNA Complex

Good water solubility, large surface area, and convenient chemical modification make graphene oxide (GO) an interesting and unique nanomaterial. Recently, GO has shown great potential in bioanalysis applications, which benefits from its outstanding biocompatibility, as well as its strong but dynamic interactions with biomolecules. DNA forms complicated binding interactions with GO, including the stacking between the DNA bases and the hydrophobic domains of GO as well as the hydrogen bonding and the electrostatic repulsion with the oxygen‐rich domains.^[^ ^162^ ^]^ The following factors affect the interaction between DNA and GO:^[^ ^163^ ^]^ 1) The interaction will be greatly affected by the DNA sequence, i.e., purine bases (A and G) show stronger binding affinity compared with the pyrimidines (T and C). 2) Furthermore, a shorter DNA strand can be more rapidly and tightly adsorbed by GO.^[^ ^162^ ^]^ 3) There is also electrostatic repulsion between DNA and GO; thus, higher salt concentrations can screen charges and promote DNA adsorption.^[^ ^164^ ^]^ 4) Likewise, in the acidic buffers, the surface charge of GO is neutralized, and consequently, its repulsion toward DNA will be reduced, resulting in an enhanced attractive interaction. For instance, Liu et al. reported that GO absorbed less DNA at high pH value.^[^ ^165^ ^]^ 5) Temperature is also an important factor that will affect the interaction between GO and DNA, and Liu et al. observed that the faster DNA adsorption will happen at relatively higher temperatures.^[^ ^166^ ^]^

**Figure 15:**
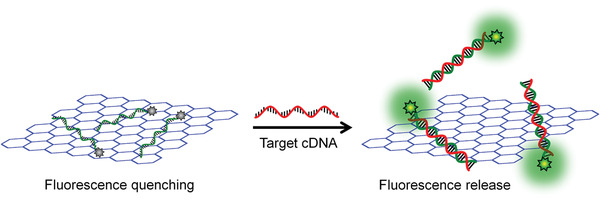
Illustration of GO‐based DNA sensor. Fluorophore‐labeled ssDNA is adsorbed by GO and its fluorescence is quenched; after forming a dsDNA with complementary target DNA, it is desorbed from GO and release the fluorescence, achieving the fluorescence‐based DNA detection.

Unpaired bases in ssDNA strongly interact with GO surface through hydrophobic interaction and *π*–*π* stacking, on the contrary, GO shows relatively low affinity to dsDNA due to the electrostatic repulsion. Therefore, GO can more efficiently adsorb ssDNA compared with dsDNA. Considering its capability to sensitively discriminate between ssDNA and dsDNA, as well as its super and universal fluorescence quenching ability, GO has been extensively used in DNA‐based fluorescence biosensors for the detection of biomolecules, metal ions, and small molecules. The working principle of GO–DNA nanosensors based on physisorption is as follows (**Figure** **15**). The fluorophore‐labeled ssDNA is adsorbed on the surface of GO, and the fluorescence is quenched by GO at this time. When the complementary target DNA is treated, the ssDNA forms double helix structures and dissociate from the GO surface; thus, the fluorophore will emit fluorescence to realize the detection of the target DNA.

Although simple to be constructed, the GO–DNA nanosensors based on physisorption remained some drawbacks. First, as the interaction between GO and DNA is sensitive to pH, it is hard to develop pH sensors based on this method.^[^ ^167^ ^]^ Second, there will be a severe specificity problem when the physisorbed sensor is applied to intracellular detection. As the intracellular environment contains a high concentration of nucleic acids and proteins, the physically adsorbed DNA might be displaced by the nontarget molecules. Therefore, GO–DNA nanosystems constructed via chemical conjugation was demonstrated to show a higher specificity for extra‐ and intracellular applications. However, both the conjugation process and the subsequent sensing process will be affected by the high affinity of the GO surface to DNA. To solve this problem, we developed a treatment method using herring sperm DNA (HSD), by which the physical adsorption from GO was weakened. Thus, the conjugation yield of DNA and the sensing specificity of the resultant GO‐DNA nanosystem were significantly improved. pH‐Sensitive GO‐DNA nanosensors have been successfully fabricated through combining the chemical conjugation and HSD passivation method, which showed excellent sensing ability not only in vitro but also in intracellular pH detection.^[^ ^168^ ^]^ Also, by the same strategy, GO–DNA nanosensors for the detection of intracellular microRNAs were fabricated, which can simultaneously discriminate among three miRNAs in live cells as well as monitor the dynamic expression of these miRNAs.^[^ ^169^ ^]^

#### 4.1.2 AuNP–DNA Complex

AuNP is one of the most important nanomaterials in the biomedical field due to its low cytotoxicity, high biostability, and distinct optical properties.^[^ ^170^ ^]^ AuNPs can be easily prepared by the reduction reaction of chloroauric acid (HAuCl_4_), and the particle size can be controlled by adjusting the concentration of citrate. Due to the localized surface plasmon resonance (LSPR) effect, AuNPs show colorimetric changes as varying from the dispersed state to the aggregated state. Also, AuNPs are efficient quenchers, which show distance‐dependent fluorescence quenching. Based on these two exciting properties, AuNPs have been extensively applied in biological analysis and detections; and AuNPs are often accompanied by DNAs in the design of sensing systems. Thiol‐modified DNA can be chemically conjugated to the surface of AuNPs through covalent bonds (Au–S chemical bonds) to form stable AuNP–DNA probes. Nonetheless, the interaction of unmodified DNAs with AuNPs also attracted a lot of research attention, as this will result in a simpler, more convenient, faster, and cost‐friendly method for the preparation of AuNP/DNA‐based biomaterials.^[^ ^163^ ^]^

There is still controversy about the driving forces of the physical adsorptions between DNAs and AuNPs. Li et al. proposed that the adsorption of ssDNA onto AuNPs was mainly maintained by electrostatic force through Derjaguin–Landau–Verwey–Overbeek (DLVO) theory.^[^ ^171^ ^]^ However, Nelson et al. believed that the hydrophobic interaction was the main interaction between DNAs and AuNPs, and they deduced the conclusion from the following phenomenon.^[^ ^172^ ^]^ First, the affinity of AuNP to ssDNA is much larger than that to dsDNA, which cannot be explained by the Debye screening as there is only a linear difference in charge density from ssDNA to dsDNA. Also, the interaction between DNA and AuNP is determined by the sequence of DNA and the types of salts in the buffer, which cannot be explained by DLVO theory but can be well described by hydrophobic interactions.

**Figure 16:**
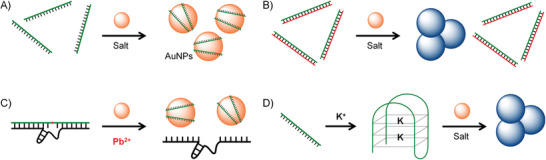
Label‐free biosensors based on the ssDNA–AuNPs complex. A,B) ssDNA can prevent the aggregation of AuNPs while dsDNA cannot. C) A colorimetric sensor for Pb^2+^ detection was designed by using the Pb^2+^‐related DNAzyme. D) A sensing system could be designed by using conformational changes of DNA in the presence of the specific analytes.

Because AuNPs can sensitively and specifically discriminate ssDNA from dsDNA, biosensors have been developed based on this principle. Owing to the high absorption coefficients, the color of AuNPs can be visually observed when the concentration is down to the nm level, which is also very sensitive to the salt concentrations.^[^ ^173^, ^174^ ^]^ Under a high concentration of salts, AuNPs can be stabilized by ssDNA against aggregation (**Figure** **16**A). In the presence of cDNA, the surface‐absorbed ssDNA can be recognized to form dsDNA, which will dissociate from the surface of AuNPs due to the significantly decreased hydrophobic interaction (all the hydrophobic bases are locked in the duplex structure), resulting in the aggregation of AuNPs and the subsequent colorimetric variations (Figure 16B). For example, the colorimetric detection of lead ions was carried out by this principle (Figure 16C). DNAzyme with a double‐stranded structure cannot prevent the aggregation of AuNPs at first; when it went through the catalyzed cleavage reaction in the presence of Pb^2+^, ssDNA as the products of cleavage could prevent salt‐induced aggregation of AuNPs, inducing colorimetric changes. In another example, in the presence of K^+^, the G‐rich ssDNA underwent conformational changes to form a folded G‐quadruplex structure. As a result, the salt‐induced AuNPs aggregation occurred, enabling the label‐free detection of K^+^ (Figure 16D).

### 4.2 Complex between DNA and Cationic Polymer

Noncovalent interactions between cationic polymers and NAs have been widely applied in biomedical fields, such as gene delivery and NA detection.^[^ ^175^ ^]^ However, the physical interaction of cationic polymers with NAs still needs further clarification. Although electrostatic forces can be intuitively determined between cationic polymers and negatively charged nucleic acids, recent studies have shown that hydrophobic forces also play an important role. In the following part, we will discuss the role of hydrophobic forces in the cationic‐polymer/DNA complexes and their biomedical applications by using two cationic polymers as examples.

#### 4.2.1 PEI–DNA Complex

Gene‐delivery is always a big challenge for gene therapy. Naked DNA or RNA generally shows poor cellular uptake capability and low biological stability; therefore, it is difficult to exert their biological effects and requires a suitable delivery system to overcome these physiological obstacles.^[^ ^14^ ^]^ Cationic polymers, as the most commonly used nonviral vector delivery systems, especially PEI, have been widely used for intracellular delivery of therapeutic NAs. However, the efficiency of PEI‐based gene delivery is not ideal due to its low efficiency in complexing with DNA and the undesired cytotoxicity.^[^ ^176^ ^]^ In order to solve this problem, chemical modifications to PEI have been performed to enhance the gene delivery efficiency through enhancing the hydrophobic interactions.

**Figure 17:**
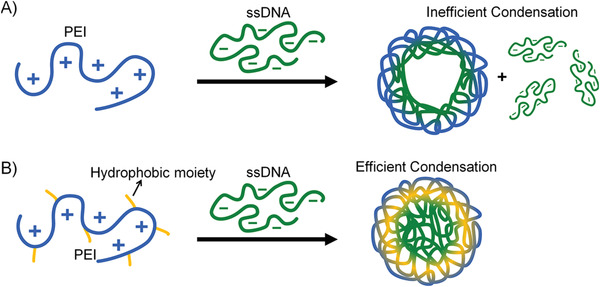
A) Complexes were formed by PEI and ssDNA through the electrostatic interaction. B) The stability of the complex between PEI and ssDNA can be enhanced by the inducing of hydrophobic interactions through the modification of hydrophobic moieties on the PEI chain.

The introduction of the hydrophobic moiety can be achieved through the following strategies.^[^ ^177^, ^178^ ^]^ Modifying the hydrophobic moieties on the PEI; or modifying the hydrophobic moieties on the NAs. The introduction of hydrophobic moieties provides the additional driving force, hydrophobic interaction, to the complexation, which makes the PEI/DNA complex easier to form and more stable (**Figure** **17**). The PEI/DNA complex with the enhanced hydrophobic interactions exhibits many excellent properties, for example, the higher DNA condensation efficiency, less cytotoxicity due to the less PEI usage, stronger biological stability, and better cell uptake capability due to the hydrophobicity‐mediated cell endocytosis.

#### 4.2.2 *π*‐Conjugated‐Polyelectrolyte–DNA Complex

*π*‐Conjugated polymers have long‐range delocalized electrons on their backbone, thereby showing many excellent properties.^[^ ^179^ ^]^ Compared with small molecule analogs, *π*‐conjugated polymers exhibit stronger light‐harvesting capabilities. Besides, the exciton can rapidly migrate along the backbone of the *π*‐conjugated polymer, achieving an efficient energy transfer to the low‐energy receptors, which is also known as the “molecular wire” effect. These two advantages make *π*‐conjugated polymers a promising candidate for the development of biosensors. However, if the *π*‐conjugated polymer is to be applied in the biological environment, it needs to be chemically modified to achieve proper water solubility. Cationic groups are generally introduced into the side chains to prepare water‐soluble cationic *π*‐conjugated polyelectrolytes.

**Figure 18:**
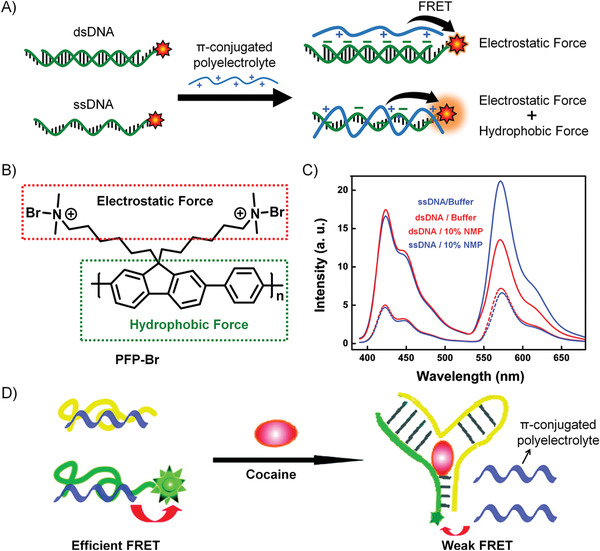
A) ssDNA shows stronger interaction with *π*‐conjugated polyelectrolytes than dsDNA because of the hydrophobic interactions. B) The aromatic ring of the *π*‐conjugated polyelectrolyte backbone provides hydrophobic forces, and the cationic side chain provides electrostatic interactions. C) More efficient FRET can be achieved from fluorescent *π*‐conjugated polyelectrolytes to the fluorophore‐modified ssDNA compared with dsDNA. D) The illustration of a sensor for cocaine detection based on *π*‐conjugated polyelectrolytes/DNA complex. The panels (C) and (D) are reproduced with permission.^[^ ^180^ ^]^ Copyright 2010, American Chemical Society.

Related studies have revealed that, except the electrostatic attraction, hydrophobic interaction between ssDNA and the backbones of the cationic *π*‐conjugated polyelectrolytes is also essential. Xia et al. found that the cationic *π*‐conjugated polyelectrolyte showed different affinities to ssDNA and dsDNA (**Figure** **18**A).^[^ ^180^ ^]^ The exposed bases of ssDNA can be complexed with the hydrophobic backbone of the *π*‐conjugated polyelectrolyte through hydrophobic interactions. While the bases of dsDNA are shielded, so the interaction between dsDNA and the polyelectrolyte becomes weaker, which was determined by FRET between the fluorophore on the DNA and the *π*‐conjugated polyelectrolyte (Figure 18B,C). Biosensors can be designed based on this principle. As shown in Figure 18D, in the absence of the target (for example, cocaine), the *π*‐conjugated polyelectrolyte strongly binds to the ssDNA; therefore, there is an efficient FRET from the polyelectrolyte to the fluorophore‐labeled on the ssDNA. When the target is present, the ssDNA is recognized to form dsDNA, which shows a relatively weaker affinity to the *π*‐conjugated polyelectrolyte, resulting in an inhibited FRET.

### 4.3 Complex between Structural DNA Self‐Assembly and DNA Amphiphiles

Base pairing of nucleic acid is the central principle of DNA self‐assembly. Since Seeman used DNA as a building block to construct nanomaterials since the 1980s, a variety of DNA self‐assemblies have been developed, such as DNA origami, framework nucleic acid, and DNA cage.^[^ ^17^ ^]^ Moreover, due to biocompatibility and diverse biological functionalities, these DNA self‐assemblies have been widely applied in biomedical fields, including but not limited to drug delivery, gene regulation, immune regulation, and biosensors.^[^ ^18^ ^]^ However, nanostructures formed solely by the hydrogen‐bonding of base‐pairing typically require hundreds of DNA strands, which results in the high cost and the complexity in operation. Therefore, it is necessary to introduce other self‐assembly driving forces to enrich the diversity of nanostructures and simplify the preparation of DNA‐based nanomaterials.

Introducing hydrophobic interactions will increase the diversity and functionality of DNA nanomaterials. DNA amphiphiles show distinct interface properties, i.e., the DNA block can be used for the programmable molecular‐hybridization, and the hydrophobic moiety provides the hydrophobic interaction. Therefore, DNA amphiphiles can be considered as the best candidates to introduce hydrophobic interactions into the design of novel DNA nanomaterials.

#### 4.3.1 DNA Origami–DNA Amphiphiles

DNA origami is a technique that folds long ssDNA into the desired structure with the help of hundreds of short‐stranded DNAs, in which the long single strand is called a scaffold, and the short strands are called staples. Nanostructures with exact dimensions can be prepared by the DNA origami technique, including smiley faces, vases, and pentagrams.^[^ ^19^ ^]^ However, it is hard to construct macroscopic structures only by the hydrogen‐bonding of base pairing in DNA origami technique. Introducing additional hydrophobic interactions is a practical approach to solve this problem, and by which more functionalities are possible to be incorporated.

**Figure 19:**
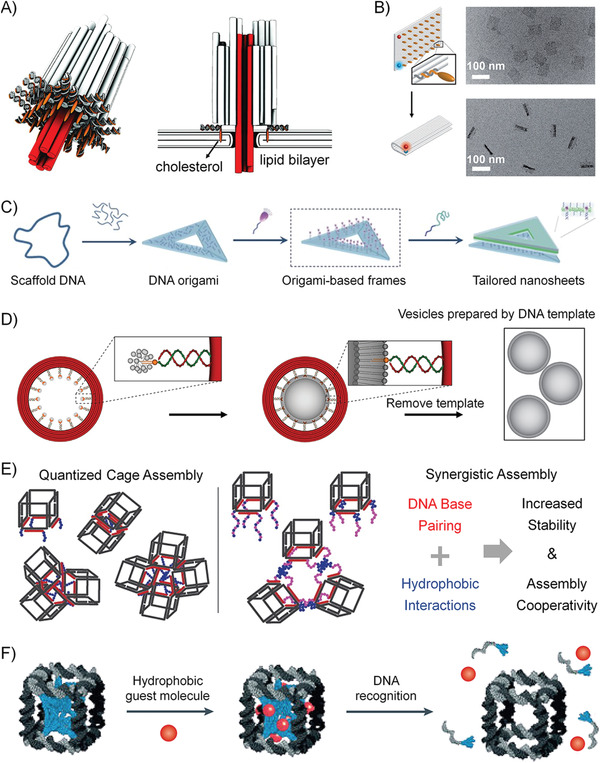
A) Illustration of the artificial transmembrane channels prepared by DNA origami technique. The cylinder represents the DNA double helix structure. The red is the stem inserted into the cell membrane. The orange is cholesterol‐modified DNA used to anchor this artificial ion channel into the lipid bilayer. Reproduced with permission.^[^ ^181^ ^]^ Copyright 2012, AAAS. B) Driven by hydrophobic forces, a single layer of DNA origami is folded into a double‐layered structure. The TEM images show the DNA origami sheets before and after folding. Reproduced with permission.^[^ ^182^ ^]^ Copyright 2014, Wiley‐VCH. C) Hydrophobic molecules are hybridized to DNA origami through DNA tail to form a hydrophobic framework, which can self‐assemble into higher‐order structures. Reproduced with permission.^[^ ^183^ ^]^ Copyright 2016, Wiley‐VCH. D) DNA origami was used as a template to mediate the formation of vesicles with the controlled size and shape. Reproduced with permission.^[^ ^185^ ^]^ Copyright 2016, Nature Publishing Group. E) Different quantized cage assemblies controlled by the length of the hydrophobic polymer; DNA cage‐loop structures can be prepared by adjusting the hydrophobicity of the DNA amphiphiles. Reproduced with permission.^[^ ^186^ ^]^ Copyright 2016, American Chemical Society. F) The hydrophobic polymer is packed into a DNA cage by molecular hybridization. The hydrophobic environment in the cage can contain the guest molecule and release the guest molecule in the presence of specific DNA sequence. Reproduced with permission.^[^ ^187^ ^]^ Copyright 2013, Nature Publishing Group.

Simmel et al. used DNA origami to prepare a hollow stem, on which lipid membranes were inserted to fabricate the columnar channels through the cholesterol anchors. The as‐prepared nanostructure behaved as the artificial nanoscale transmembrane channel, which showed a response similar to that of natural ion channels (**Figure** **19**A).^[^ ^181^ ^]^ In another work, Simmel et al. introduced cholesterol‐modified DNA into the monolayered DNA origami structures by chain hybridization. Because the hydrophobic part of the DNA amphiphile has a strong tendency to aggregate in an aqueous solution, the single‐layer DNA origami structure with cholesterol folded into a sandwich‐like double‐layer structure due to the hydrophobic interactions (Figure 19B). This bilayer structure could also be expanded to multiple layers by adding a surfactant or a phospholipid bilayer membrane.^[^ ^182^ ^]^

Generally, the amphiphilic molecules tend to self‐assemble into 3D spherical structures rather than the 2D nanostructures in aqueous solution. Liu et al. used the DNA origami structure as a 2D template to rivet the DNA amphiphiles by DNA hybridization. Through hydrophobic interactions, a continuous nanosheet was assembled from DNA amphiphiles in an aqueous solution (Figure 19C).^[^ ^183^, ^184^ ^]^ Similarly, controlling the shape and size of vesicles at the nanoscale is challenging, and achieving this goal may require careful optimization of lipid composition and manufacturing processes. Lin et al. used DNA origami to prepare a circular DNA template, and then hybridized the lipid‐modified DNA to the origami template, which could further mediate the formation of vesicles with the expected sizes and shapes (Figure 19D).^[^ ^185^ ^]^

#### 4.3.2 DNA Cage–DNA Amphiphiles

DNA cages can be assembled from only a few DNA strands through complementary base pairing. However, it is challenging to prepare a large number of complex nanostructures with only a few DNA strands from only one self‐assembly language (base pairing).^[^ ^188^ ^]^ Sleiman et al. hybridized DNA amphiphiles to DNA cages. The hydrophobic block of the amphiphile on the side of the cage can induce aggregation of the DNA cages,^[^ ^186^, ^189^ ^]^ and the degree of aggregation can be well controlled by the length of the hydrophobic block of the DNA amphiphile. Also, DNA cage‐loop structures can be prepared by adjusting the hydrophilic/hydrophobic ratios of DNA amphiphiles (Figure 19E).^[^ ^186^ ^]^ Besides, the hybridization of hydrophobic polymers on both sides of a DNA cage results in a “handshake” of the polymers within the cage, resulting in a DNA micelle cage. Therefore, they further prepared monodisperse crosslinked polymer nanoparticles in DNA cages, the hydrophobic environment of which can be used to load guest molecules; and the guest molecules can be released by the presence of specific DNA sequences (Figure 19F).^[^ ^187^ ^]^

### 4.4 Complex between DNA Amphiphiles and Some Nanoarchitectures

The self‐assemblies of common block copolymers have been widely studied, such as spherical micelles, vesicles, sheets, tubes, which show a large number of applications in the field of materials science and biomedicine. Unlike the base‐pairing language of DNA, the self‐assembly of classic block copolymers is governed by the interactions including, electrostatic forces, hydrophobic interactions, and *π*–*π* stacking.^[^ ^32^ ^]^ The incorporation of the specific hybridization properties of DNA into the self‐assembly of the typical block copolymers will produce incredible assembly structures and satisfy some specific applications.

#### 4.4.1 Micelle–DNA Amphiphile Complex

**Figure 20:**
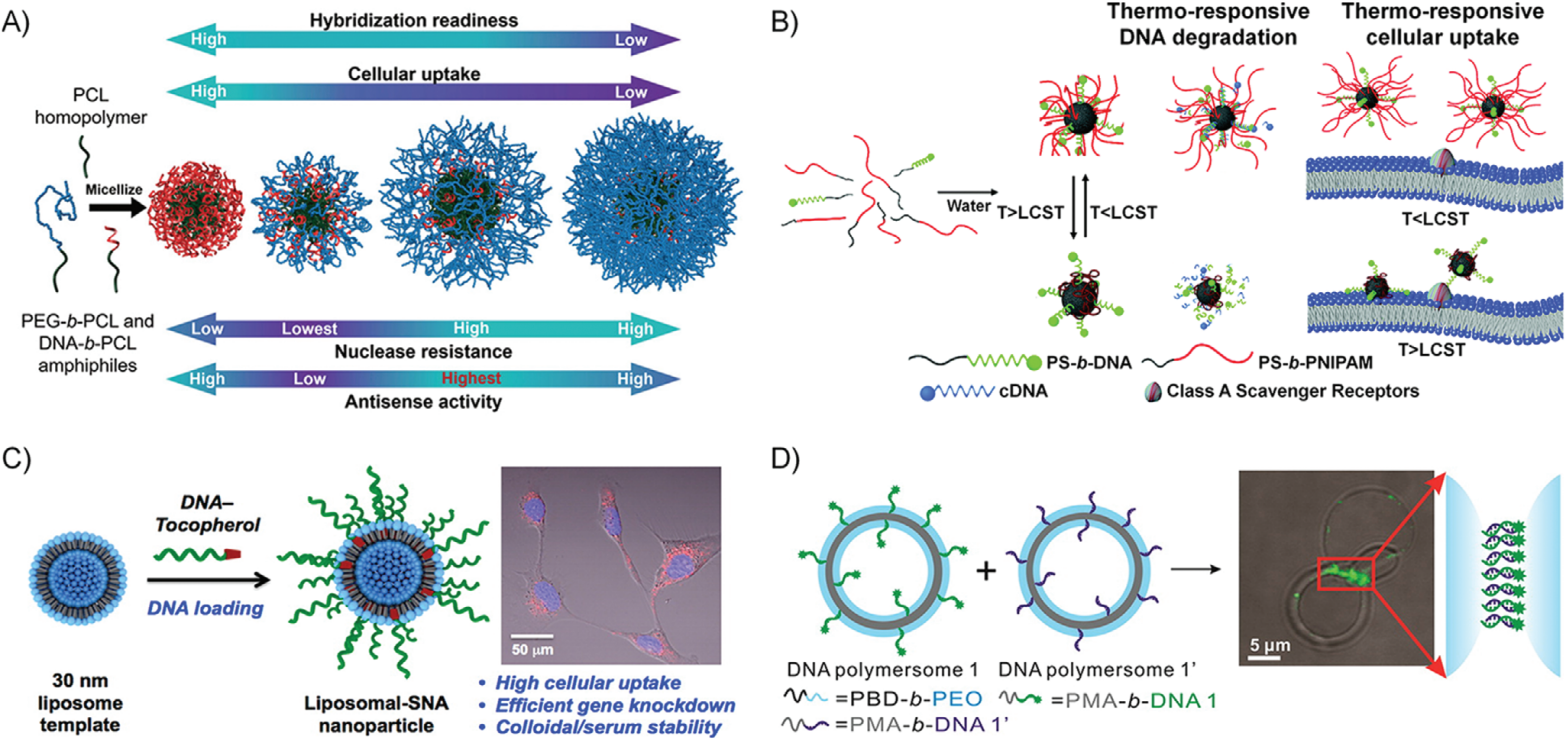
A) PEG‐*b*‐PCL and DNA‐*b*‐PCL can be coassembled into mixed micelles. Reproduced with permission.^[^ ^71^ ^]^ Copyright 2017, American Chemical Society. B) Thermal‐responsive cell‐uptake of PNIPAM/DNA hybrid micelles. Reproduced with permission.^[^ ^193^ ^]^ Copyright 2019, Royal Society of Chemistry. C) The DNA‐tocopherol amphiphiles can be inserted into the phospholipid bilayer of liposome through hydrophobic interactions to form liposomal SNAs, which show higher cellular uptake, more efficient gene knockdown, and higher biostability. Reproduced with permission.^[^ ^194^ ^]^ Copyright 2014, American Chemical Society. D) Binary coassembly of PBD‐*b*‐PEO and PMA‐*b*‐DNA into giant mixed polymersomes; and the imaging of the formation of DNA islands at the junction site of two polymersomes. Reproduced with permission.^[^ ^195^ ^]^ Copyright 2016, American Chemical Society.

Classical amphiphilic block copolymers form small‐sized micelles in aqueous solutions at specific hydrophilic‐hydrophobic ratios.^[^ ^190^ ^]^ Its inner core is hydrophobic, and its shell is hydrophilic, which has been widely used in drug and gene delivery.^[^ ^31^, ^191^ ^]^ Through the coassembly method, the PEG‐based amphiphile and the DNA‐based amphiphile can form a mixed micelle together, and the DNA in this mixed micelle still retains the ability of hybridization.^[^ ^192^ ^]^ Zhang et al. coassembled the PEG‐*b*‐PCL amphiphilic block copolymer with DNA‐*b*‐PCL to prepare mixed micelles and studied in detail the effect of the ratios of PEG/DNA on DNA properties, including the hybridization dynamics, cellular uptake, biostability, and gene regulation capability (**Figure** **20**A).^[^ ^71^ ^]^ Park et al. reported that PS‐*b*‐DNA and PS‐*b*‐PNIPAM were coassembled into a mixed micelle with a PS core and a DNA/PNIPAM mixed shell (Figure 20B).^[^ ^193^ ^]^ Due to the temperature‐responsive properties of PNIPAM, when the temperature is higher than the lower critical solution temperature (LCST), PNIPAM would change from a hydrated coil to a dehydrated collapsed state. As a result, the DNA strands in the shell of mixed micelle would be exposed, showing faster DNA degradation rates and better cell uptake through receptor‐mediated endocytosis. This dynamically switchable DNA recognition properties of DNA based nanostructure offers new opportunities for the design of intelligent drug delivery systems in the future.

#### 4.4.2 Vesicle–DNA Amphiphile Complex

Amphiphilic molecules can also self‐assemble into a vesicle structure in an aqueous solution under a specific hydrophilic/hydrophobic ratio, which has a hollow aqueous cavity and a lipophilic bilayer.^[^ ^196^ ^]^ In one of the examples, DNA amphiphiles conjugated with tocopherol tails were anchored into the phospholipid layer of the liposomes, which were self‐assembled from 1,2‐dioleoyl‐*sn*‐glycerol‐3‐phosphate choline (DOPC), resulting in liposome SNAs (Figure 20C).^[^ ^194^ ^]^ This liposome SNA has a high‐density of DNAs on the surface and displays typical “SNA” properties, such as the robust biological stability and the highly efficient cell internalization. Also, the properties of the vesicles are related to the sequence of DNA, e.g., the fusion between vesicles can be promoted by the presence of DNA.^[^ ^197^, ^198^, ^199^ ^]^ Alternatively, DNA amphiphiles can be used to regulate cell‐to‐cell interactions through the anchoring of hydrophobic moieties and molecular hybridization of DNA.^[^ ^200^, ^201^, ^202^ ^]^ In another work, Park et al. reported that two amphiphilic block copolymers, poly(butadiene)‐block‐poly(ethylene oxide) (PBD‐*b*‐PEO) and polymethyl acrylate‐block‐DNA (PMA‐*b*‐DNA), could coassemble to form giant polymersomes (Figure 20D); when two of this kind of polymersomes interacted via strand‐hybridization, the DNA that originally uniformly distributed on the polymersome would gather to one side and form a DNA island.^[^ ^195^ ^]^

#### 4.4.3 2D Nanoassembly–DNA Amphiphile Complex

**Figure 21:**
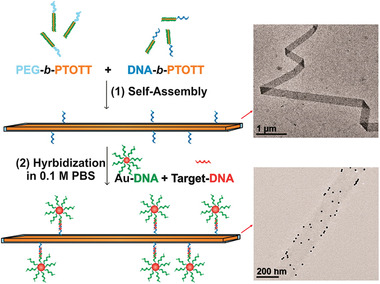
Scheme of DNA‐*b*‐PT and PEG‐*b*‐PT coassembly into DNA‐functionalized 2D nanoribbons. On the right are the TEM images of the DNA‐functionalized nanoribbons before and after the modification with AuNPs. Reproduced with permission.^[^ ^81^ ^]^ Copyright 2014, American Chemical Society.

In addition to common micelle and vesicle structures, amphiphilic block copolymers can also self‐assemble into other unusual architectures, such as cylinders and sheets.^[^ ^33^ ^]^ In particular, amphiphilic conjugated polymers will exhibit different self‐assembly properties, due to their special characteristics, such as *π*–*π* stacking and the rigid molecular‐structures. Park et al. prepared an amphiphilic conjugated polymer, PEG‐*b*‐PT (polythiophene). PEG‐*b*‐PT can self‐assemble into a variety of morphologies in aqueous solutions, including flakes, vesicles, and nanoribbons.^[^ ^203^ ^]^ Thereafter, they used DNA‐*b*‐PT to coassemble with PEG‐*b*‐PT at a molar ratio of 1:200 to form DNA‐functionalized nanoribbons, and the DNA hybridization ability on the nanoribbons was verified by the presence of cDNA‐modified AuNPs, which can be observed by TEM imaging (**Figure** **21**).^[^ ^81^ ^]^

## 5 Conclusion and Perspective

DNA‐based biomaterials exhibit unparalleled potential in structural design and functional applications. Compared with other building blocks, like peptides or synthetic polymers, DNA shows many sequence‐dependent functionalities and can realize the precise nanostructure construction through the exact base‐pairing interactions. Besides, as one of the most basic driving forces in nature, the introduction of hydrophobic interactions has expanded the scope of DNA‐based biomaterials and reduced their preparation costs. Moreover, DNA self‐assemblies driven by hydrophobic interactions exhibit stronger biological stability and contain hydrophobic domains for the loading of functional molecules. Therefore, hydrophobic interactions play a more and more important role in the development of DNA‐based biomaterials.

According to the discussions in this review, utilizing hydrophobic interaction to fabricate DNA‐based biomaterials shows several advantages over the other strategies, and the application prospects are also discussed. 1) The phase separation of long strand DNA provides an efficient and low‐cost method for the fabrication of DNA‐based biomaterials. In this system, DNA can be used not only as delivery carriers but also as functional cargoes (e.g., siRNA, antisense DNA, and DNAzyme). Additionally, traditional chemotherapeutics (e.g., doxorubicin) can be loaded into the DNA nanoparticles. Hence, this strategy can be applied to drug delivery and gene regulation. Besides, stimuli‐responsive DNA sequences (e.g., pH and ions) can also be integrated into the DNA nanoparticles, achieving smart drug delivery. Other materials can also be introduced into this system for increasing functionalities, such as silver nanoclusters for optical properties and gold nanoparticles for heat effect. 2) As for DNA amphiphiles, many organic functional molecules can be introduced to enrich the functionalities of the resultant self‐assemblies, such as fluorescence dyes, organic semiconducting molecules with photothermal or photodynamic capabilities, or stimuli‐responsive polymers. The assembly formed by DNA amphiphiles contains a hydrophobic domain that can accommodate some functional materials for special applications, such as NIR‐II dye and magnetic resonance contrast agents. 3) The dynamic and highly tunable hydrophobic interactions will make the resultant materials more sensitively respond to the environmental stimuli, providing a new avenue to develop smart biomaterials. For instance, as the aggregation‐state of the DNA amphiphiles can be well controlled by the hydrophilic DNA block, organic dyes are used as the hydrophobic blocks, and some innovative DNA‐based biosensors have been developed. When the DNA block recognizes targets by chain hybridizations or conformational changes, the amphiphilicity of the self‐assembly system will be altered to result in further aggregation of the DNA amphiphiles or dissociation of the self‐assembled micelles. Therefore, the aggregation states of the organic dyes will be changed to show significantly different optical properties. In another example, due to the hydrophobic interactions, DNA amphiphiles showed more efficient albumin binding. Thus, by the conjugation with the hydrophobic lipid, the CpG‐lipid amphiphiles showed a 30‐fold increase in T‐cell priming and the significantly enhanced immunotherapy efficacy. 4) Hydrophobic interaction offers an efficient way to construct higher‐order self‐assembly structures. DNA origami is the most potent nanotechnology, which can create almost any arbitrary shapes with precisely defined dimensions at the nanoscales. However, one of the major challenges of the DNA origami technique is the construction of macroscopic structures only by the hydrogen‐bonding of base pairing. Introducing additional hydrophobic interactions is an effective approach to solve this problem. The nanosized building blocks created by the DNA origami technique are possible to be further organized by DNA amphiphiles to self‐assemble into higher‐order bulky structures. On one hand, as the hydrophobicity of the hydrophobic block can be easily tuned, a variety of higher‐level self‐assembled structures are possible to be realized. On the other hand, the hydrophobic blocks can bring in more functionalities to the final materials. 5) Clarifying the rules of controlling and utilizing hydrophobic interactions will promote the development of new DNA‐based functional materials. As discussed in the second part, DNA itself shows thermal‐responsive properties, which will change from the dispersed state to the aggregated state at a certain temperature driven by the hydrophobic interaction. Walther et al. revealed that this thermal‐controlled phase separation properties of DNA are dependent on the composition and the polymerization degree of the DNA chains. Based on their observations, they could construct hierarchical self‐assembled architectures by the sophisticated design of the hydrophobic interactions, which also showed temperature‐controlled cargo release capability. For the DNA/inorganic nanoparticle complexes formed by hydrophobic interactions, it also provides a nonlabeled way to fabricate multifunctional materials. 6) The application field of the DNA‐based materials has been significantly extended by the comprehensive understanding and widespread use of hydrophobic interactions. For example, amphiphilic DNA molecules can be used to regulate the interaction between different cells, which provides great possibilities for cell engineering research, such as cell signal transduction, cell microenvironment regulation, and cell presentation. Also, amphiphilic DNA may show excellent potential in cell‐based therapies, such as regulating immune cells or other circulating cells. In terms of synthetic biology, DNA‐based biomaterials combined with hydrophobic interactions may facilitate the preparation of artificial organelles and even artificial cells.

Although DNA‐based biomaterials combined with hydrophobic interactions exhibit many excellent properties, there are still some challenges that need to be overcome, both in the design of functional materials and in practical clinical transformation. First, the precise control of the degree of hydrophobic interactions in pure DNAs, DNA amphiphiles, and DNA complexes is still an unresolved issue. More systematic studies on the DNA‐based materials are still required to give the structure‐hydrophobic interaction‐property relationship. Also, although nanoscale DNA‐based biomaterials exhibit improved biological stability compared with free DNA, minimizing the degradation of ribozymes is still a challenge, especially in vivo. Due to the immunogenicity of DNA itself, the safety of DNA‐based biological materials in vivo still needs to be carefully evaluated before the clinical transformation. Hydrophobic interaction is a weak interaction, which will be affected by many factors, such as solution dilution and interference by other amphiphilic molecules; therefore, how to protect this DNA‐based biomaterial based on hydrophobic interaction is also a challenge. Moreover, amphiphilic DNA will tend to be inserted into the cell membrane through hydrophobic interactions, which may cause unpredictable cell damage. As for the strategy where DNA and other materials complex through hydrophobic interaction, although these composite materials have exhibited great potential in various biological applications, their properties still need to be further explored.

In summary, DNA‐based biomaterials combined with hydrophobic interaction are a very comprehensive and extensive platform. In this platform, various functional or structural DNA can be easily introduced; and the hydrophobic interaction provides the driving force for self‐assembly, which allows these DNA‐based materials to show diversified functionalities, more sensitive stimuli‐responsiveness, hierarchical self‐assembly capability, and more complicated interactions with biological molecules or cells. Therefore, we believe that the integration of DNA properties and hydrophobic interactions will become a trend to address the practical problems that need to be solved in the biomedical field, and hydrophobic interaction will become a necessary tool in the design of DNA‐based biomaterials in the future.

## Conflict of Interest

The authors declare no conflict of interest.
